# Methyl Jasmonate and 1-Methylcyclopropene Treatment Effects on Quinone Reductase Inducing Activity and Post-Harvest Quality of Broccoli

**DOI:** 10.1371/journal.pone.0077127

**Published:** 2013-10-16

**Authors:** Kang Mo Ku, Jeong Hee Choi, Hyoung Seok Kim, Mosbah M. Kushad, Elizabeth H. Jeffery, John A. Juvik

**Affiliations:** 1 Department of Crop Sciences, University of Illinois at Urbana-Champaign, Urbana, Illinois, United States of America; 2 Department of Food Science and Human Nutrition, University of Illinois at Urbana-Champaign, Urbana, Illinois, United States of America; 3 The Distribution System Research Group, Korea Food Research Institute, Gyeonggi-do, South Korea; University of South Florida College of Medicine, United States of America

## Abstract

Effect of pre-harvest methyl jasmonate (MeJA) and post-harvest 1-methylcyclopropene (1-MCP) treatments on broccoli floret glucosinolate (GS) concentrations and quinone reductase (QR, an *in vitro* anti-cancer biomarker) inducing activity were evaluated two days prior to harvest, at harvest and at 10, 20, and 30 days of post-harvest storage at 4 °C. MeJA treatments four days prior to harvest of broccoli heads was observed to significantly increase floret ethylene biosynthesis resulting in chlorophyll catabolism during post-harvest storage and reduced product quality. Post-harvest treatment with 1-methylcyclopropene (1-MCP), which competitively binds to protein ethylene receptors, maintained post-harvest floret chlorophyll concentrations and product visual quality in both control and MeJA-treated broccoli. Transcript abundance of *BoPPH*, a gene which is responsible for the synthesis of pheophytinase, the primary enzyme associated with chlorophyll catabolism in broccoli, was reduced by 1-MCP treatment and showed a significant, negative correlation with floret chlorophyll concentrations. The GS, glucobrassicin, neoglucobrassicin, and gluconasturtiin were significantly increased by MeJA treatments. The products of some of the GS from endogenous myrosinase hydrolysis [sulforaphane (SF), neoascorbigen (NeoASG), *N*-methoxyindole-3-carbinol (NI3C), and phenethyl isothiocyanate (PEITC)] were also quantified and found to be significantly correlated with QR. Sulforaphane, the isothiocyanate hydrolysis product of the GS glucoraphanin, was found to be the most potent QR induction agent. Increased sulforaphane formation from the hydrolysis of glucoraphanin was associated with up-regulated gene expression of myrosinase (*BoMyo*) and the myrosinase enzyme co-factor gene, *epithiospecifier modifier1* (*BoESM1*). This study demonstrates the combined treatment of MeJA and 1-MCP increased QR activity without post-harvest quality loss.

## Introduction

*Brassica* vegetables are recognized as functional foods that have putative cancer preventive effects as shown in epidemiological and animal carcinogenesis studies [1]. The glucosinolates (GS) including glucoraphanin, gluconasturtiin, and sinigrin found in the tissues of accessions of *Brassica oleracea* have been identified as potent cancer prevention agents because products of their hydrolysis by the endogenous enzyme myrosinase generate sulforaphane, phenethyl isothiocyanate (PEITC), and allyl isothiocyanate (AITC). These isothiocyanate products have been shown to induce phase II detoxification enzymes such as glutathione S-transferases (GSTs) and quinone reductase (QR) in *in vitro* or *in vivo* systems that can enhance detoxification and elimination of carcinogens from the human body [2-4]. QR activity elevation with *in vitro* and *in vivo* systems has been shown to correlate with induction of other protective phase II enzymes such as the GSTs and provides a reasonable biomarker for the potential chemoprotective effect of phytochemicals against initiation of carcinogenesis [5]. In addition, several hydrolysis products from other GS found in *B. oleracea* cultivars including indole-3-carbinol [6] and *N*-methoxyindole-3-carbinol [7,8] have also been reported to promote antiproliferative bioactivity in human cancer cell lines. 

GS biosynthesis genes have been intensively studied in *Arabidopsis* using biochemical assays. There is high homology of the genes in GS biosynthesis between *Arabidopsis* and *Brassicaceae* [9]. CYP79 catalyzes the conversion of amino acids to aldoximes. *CYP79F1* and *CYP79F2* genes are responsible for aldoxime metabolism leading to aliphatic GS derived from chain-elongated methionine derivatives, whereas *CYP79B2* and *CYP79B3* have distinct functions in indolyl GS biosynthesis, which is derived from tryptophan [10]. In the biosynthetic pathway of indolyl GS, *CYP79B2* gene product catalyzes the conversion of tryptophan to indole-3-acetaldoxime, with *CYP83A1* and *CYP83B1* metabolizing the phenylalanine- and tyrosine-derived aldoximes [10]. It has been reported that indolyl GS biosynthesis is increased by jasmonic (JA) treatment [11]. The biological activity of GS varies with diversity of structure of the side chain that is the last step of GS biosynthesis [12]. Recently, methoxylation genes involved in glucobrassicin biosynthesis such as *CYP81F2*, *CYP81F3, and CYP81F4* were identified by genetic engineering *Arabidopsis* indolyl GS biosynthesis into *Nicotiana benthamiana* [13]. *CYP81F2* gene product is responsible for the methoxylation of glucobrassicin resulting in 4-methoxyglucobrassicin. The hydrolysis product of 4-methoxyglucobrassicin has been reported to be antibiotic to fungal pathogens and to the green peach aphid (*Myzus persicae*) [14,15].

Intact GS do not display bioactivity but following hydrolysis by the endogenous enzyme myrosinase, isothiocynates and other products are generated, which have been associated with insect resistance and anti-cancer activity. When the plant tissue is disrupted, myrosinase and substrates (GS) come into contact, resulting in GS hydrolysis. The chemical structure of hydrolysis products depends on the structure of the GS side chain and reaction conditions such as pH, concentration of Fe^2+^ and presence of epithiospecifier protein (ESP), a myrosinase co-factor that will favor formation of nitriles [16]. In the absence of ESP, the addition of Fe^2+^ ions also promotes nitrile formation, which are essentially without anti-cancer activity compared to the isothiocyanates like sulforaphane, PEITC, and AITC [17]. The *epithiospecifier* modifier *1* (*ESM1*) gene in *Arabidopsis* encodes a protein shown to inhibit function of ESP, leading to increased isothiocyanate production from GS hydrolysis [18].

 Methyl jasmonate (MeJA), a plant signal transduction compound associated with herbivore defense, can act as an elicitor to enhance GS biosynthesis [19]. Previous research has shown that MeJA treatments can significantly increase QR inducing activity mediated by enhancement of GS biosynthesis including glucoraphanin, glucobrassicin and neoglucobrassicin in cauliflower [20]. However, 1 mM MeJA treatment was also found to significantly promote ethylene production and increased 1-aminocyclopropane-1-carboxylate acid (ACC) concentrations and ACC oxidase activity associated with senescence and loss of product quality in broccoli [21]. Thus, while MeJA treated broccoli can display enhanced QR activity associated with increased GS concentrations, elevated ethylene production can accelerate post-harvest senescence, phytochemical degradation and visual quality loss. 

 Inhibition of plant ethylene perception or blocking the ethylene receptor using 1-methylcyclopropene (1-MCP) is an effective way to improve shelf life and quality of fruits and vegetables [22] and application of 1-MCP increases shelf life of broccoli [23]. 1-MCP application has also been found to maintain the phytochemicals in broccoli such as chlorophylls, carotenoids, ascorbic acid and GS after harvest by binding to the **ethylene** receptors ETR1 and ETR2 [24-26]. Visual color is a critical factor in retailer and consumer evaluation of product quality and subsequent purchasing decisions [27]. Chlorophyll content is considered a good indicator of broccoli post-harvest quality. Previous studies reported that pheophytinase (PPH) and pheophorbide a oxygenase (PaO) are key enzymes in post-harvest chlorophyll breakdown [28,29]. It was reported that gene expression of *BoPPH* and *BoPaO* is reduced by 1-MCP treatment [30]. 

 In this experiment, we evaluate MeJA treatment on post-harvest quality and phytochemical content of broccoli to see if 1-MCP can modulate MeJA initiated postharvest senescence. Thus, the objectives of this research are to evaluate the effect of pre-harvest MeJA and post-harvest 1-MCP treatment on postharvest physicochemistry and quinone reductase bioactivity of broccoli floret extracts. 

## Materials and Methods

### Plant Cultivation and Sample Preparation with Treatments

**Figure 1:**
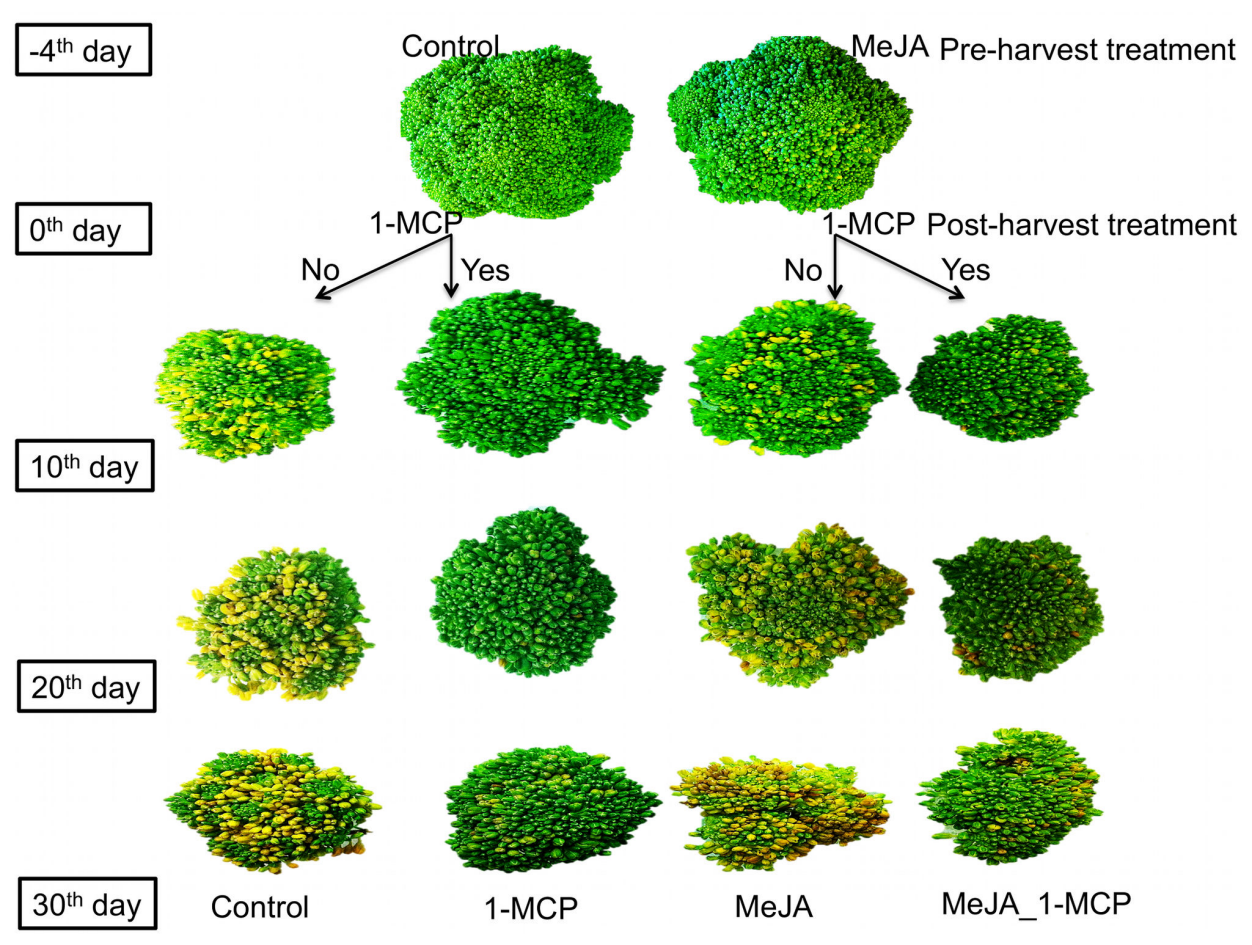
Representative samples of broccoli branchlets with or without pre-harvest MeJA and 1-MCP treatments at harvest (4 days after MeJA treatment, day 0 for 1-MCP) and at 10, 20, and 30 days of post-harvest storage at 4 °C.

‘Green Magic’ broccoli (Sakata Seed Co., Morgan Hill, CA) was used for this experiment. Broccoli seeds were germinated in 32 cell plant plug trays filled with sunshine® LC1 professional soil mix (Sun Gro Horticulture, Vancouver, British Columbia, Canada). Seedlings were grown in a greenhouse at the University of Illinois at Champaign-Urbana under a 25 °C/15 °C and 14 h/10 h: day/night temperature regime and with supplemental lighting. Forty days after seed germination, seedlings were first transferred into 1-liter pots and then after a month 150 broccoli seedlings were repotted into 3.75-L pots. These broccoli seedlings were evenly placed on three greenhouse benches and control and MeJA treatment assigned within each bench to minimize micro-environmental variation. 500 micromoles of MeJA (Sigma-Aldrich, St. Louis, MO, USA) in solution containing 0.1% ethanol was sprayed on aerial tissues of each of the treated plants four days prior to harvest at commercial maturity. Timing of MeJA sprays and concentration of solution was previously determined to optimize up-regulation of indolyl GS [31]. For the control group, only a 0.1% ethanol solution was applied. At commercial market maturity 50 broccoli heads were harvested from both the control and MeJA treated plants, transported to the laboratory, and divided into branchlets of broccoli florets. Branchlets of control and MeJA treated plants were each randomly divided into two groups generating four treatment groups: (1) No MeJA or 1-MCP (Control); (2) No MeJA and 500 ppb treatment with 1-MCP for 24 h (1-MCP); (3) MeJA without 1-MCP (MeJA), and (4) MeJA and 500 ppb treatment with 1-MCP for 24 h (MeJA_1-MCP). Treatments (1,3) and treatments (2,4) were placed in airtight plastic containers at 20 °C. 1-MCP was generated in containers holding treatments (2,4) by adding an activator and a Smartfresh^®^ tablet (AgroFresh, Inc. a division of Rohm and Hass, Philadelphia, PA, USA) to the activation solution following the instructions provided by the company. After treatment, broccoli branchlets were stored in a walk-in cooler at 4 °C. At each sampling date [-2 (2 days after MeJA treatment), at harvest (4 days after MeJA treatment, day 0 for 1-MCP), and at 10, 20, and 30 days of post-harvest storage], three random subsamples of branchlets (replications) of each treatment group were selected and assayed for ethylene and CO_2_ production and visual quality. Pictures of broccoli florets and their relative visual quality from each assay date are presented in Figure 1. After measuring CO_2_, ethylene production, and hue angle, a measurement of floret color change, a subsample of tissue from each replication was taken, frozen in liquid nitrogen, and stored at -80 °C until ground with a mortar and pestle in liquid nitrogen for RNA extraction. Residual sample tissues from each replication were freeze-dried. Freeze-dried broccoli floret tissue of each sample was finely ground with a commercial coffee grinder. The ground freeze-dried broccoli samples were stored at -20 °C prior to GS quantification and quinone reductase bioactivity assay.

### Respiration and Ethylene Production Measurement

Respiration was measured as tissue CO_2_ production. Three subsamples (300 g each) of broccoli branchlets from each treatment were placed into 3 L jars and enclosed with a silicon rubber cap for 1 h at 20 °C. Sample CO_2_ was estimated using 2% CO_2_ in a nitrogen gas (v/v) standard for each experiment. The headspace gas in the jar was sampled with a 0.2 mL plastic hypodermic syringe and injected into a GC (model Perkin Elmer AutoSystem Gas Chromatograph) equipped with a Propak® (Waters Co., Milford, MA) column and thermal conductivity detector (TCD). Temperature of the injector, detector and column was 100, 150 and 30 °C, respectively. The results were expressed as mL of CO_2_/kg/h. Ethylene measurement was measured as previously reported [20] using pure ethylene gas as a standard for estimating sample concentrations. 

### Determination of Total Chlorophyll Content

Frozen floret tissue samples from each sampling date (75 mg) were ground and extracted in 1.5 ml of 80% acetone in a 2 mL tube with vigorous vortexing for 1 h. Total chlorophyll content was determined by using the equations listed below [32]. 

Total chlorophyll (µg/mg) = 20.2 (A_645_) + 8.02 (A_663_)

Chlorophyll a (µg/mg) = 12.7 (A_663_) - 2.69 (A_645_)

Chlorophyll b (µg/mg) = 22.9 (A_645_) - 4.68 (A_663_)

### Hue Angle Measurement

As a reliable measure of color change during post-harvest storage sample, hue angle was measured as by using a LabScan XE colorimeter (Hunter Associates Laboratory, Reston, VA, USA) generating values for a* (redness and greenness), and b* (yellowness and blueness). The instrument was calibrated with a standard white and black tile. The average of four different broccoli branchlets was recorded in each replication. Hue degree (h°) was calculated as h° = tan^-1^ (b*/a*) when a*>0 and b*>0, or as h° = 180° - tan^-1^ (b*/a*) when a<0 and b>0 [20].

### Determination of Sample GS Concentrations

Freeze-dried broccoli powder (0.2 g) and 2 mL of 70% methanol were added to 10 mL tubes (Nalgene, Rochester, NY, USA) and heated on a heating block at 95 °C for 10 min. After cooling on ice, 0.5 mL benzylglucosinolate (1 mM) was added as an internal standard (POS Pilot Plant Corp, Saskatoon, SK, Canada), mixed, and centrifuged at 3,000 × g for 15 min at 4 °C. The supernatant was saved and the pellet was re-extracted with 2 mL 70% methanol at 95 °C for 10 min and the two extracts combined. A subsample (1 mL) from each pooled extract was transferred into a 2-mL microcentrifuge tube. Protein was precipitated with 0.15 mL of a 1:1 mixture of 1 M lead acetate and 1 M barium acetate. After centrifuging at 12,000 × g for 1 min, each sample was then loaded onto a column containing DEAE Sephadex A-25 resin (Sigma-Aldrich) for desulfation with arylsulfatase (*Helix pomatia* Type-1, Sigma-Aldrich) for 18 h and the desulfo-GS eluted. One hundred µL of each sample were injected on to a HPLC. Quantification of GS using high-performance liquid chromatography was performed using a previously described protocol [33].

### Measurement of Myrosinase Activity

Myrosinase activity was optimized according to previous studies [34-36]. Crude extracts were prepared by adding 0.3 g of a finely ground freeze-dried sample in 4 mL of an extraction buffer consisting of 10 mM potassium phosphate, 1 mM ethylenediaminetetraacetic acid (EDTA), 3 mM dithiothreitol (DTT) and 5% glycerol (pH 7.0) for 20 min in an ice bath [35]. The crude extracts were centrifuged at 15,000 × g for 30 min at 4 °C. To remove endogenous GS and glucose, the crude extract was filtered through an Amicon ultrafiltration cell (Millipore, Billerica, MA, USA) with a 10 KDa molecular weight cutoff [34] and washed several times at 4 °C using the same extraction buffer at pH 7.0 [35]. Fifty µL of purified extracts and 450 µL of 0.2 mM sinigrin in 33.3 mM phosphate buffer, pH 6.5 were mixed and incubated for 40 min [36]. To stop the enzyme reaction extracts were heated at 95 °C for 10 min. The release of glucose was determined by the glucose oxidase/peroxidase/ABTS method [37] using a microplate reader (Biotek Instruments, Winooski, VT, USA). Glucose concentrations were calculated using a linear standard curve. By calculating the glucose amount in aliquots of purified extracts without sinigrin, endogenous glucose levels were subtracted in purified extracts for myrosinase activity measurement. 

### Quinone Reductase (QR) Inducing Activity

For the QR assay, 75 mg of broccoli floret powder from each sample was suspended in 1.5 mL of water in the absence of light for 4 h at room temperature in a sealed 2 mL microcentrifuge tube (Fisher Scientific, Waltham, MA, USA) to facilitate GS hydrolysis by endogenous myrosinase. Slurries were then centrifuged at 12,000 × g for 10 min and supernatants were diluted to 0.25% final concentration in the QR activity assays. The QR inducing activities of different samples were determined by means of the protocol described by Prochaska and Santamaria [38]. 

### Analysis of Glucosinolate Hydrolysis Products

The extraction and analysis of isothiocyanates and other hydrolysis products was carried out according to previously published methods with some modifications [39]. Broccoli powder (75 mg) was suspended in 1.5 mL of water in the absence of light for 4 h (optimal time for hydrolysis products of indolyl GS) and 24 h (optimal time for sulforaphane and PEITC generation from glucoraphanin and gluconasturtiin) at room temperature in a sealed 2 mL microcentrifuge tube (Fisher Scientific) to facilitate GS hydrolysis by endogenous myrosinase. Slurries were then centrifuged at 12,000 × g for 5 min and supernatants was decanted into a 2 mL microcentrifuge tube. Twenty μL of butyl isothiocyanate (0.5 mg/mL) and 4-methoxyindole (1 mg/mL, Synthonix, Wake Forest, NC, USA) were added as the internal standards for isothiocyanates and hydrolysis products of indolyl GS to quantify indole-3-carbinol (I3C), NI3C, and neoascorbigen (NeoASG), respectively, with 0.5 mL of methylene chloride. NI3C and NeoASG were purified and identified by LC-MS [8,40]. Tubes were shaken vigorously before being centrifuged for 2 min at 9,600 × g. The methylene chloride layer (200 µL) was transferred into a 350 µL flat bottom insert (Fisher Scientific) in a 2 mL HPLC autosampler vial (Agilent, Santa Clara, CA, USA) for mixing with 100 µL of a reagent containing 20 mM triethylamine and 200 mM ß-mercaptoethanol (derivatization reagent) in methylene chloride. For SF and PEITC, unlike other hydrolysis products of GS measurement, 0.5 mL of fresh broccoli extracts were mixed with 0.5 mL of derivatization reagent using an orbital shaker at 220 rpm for 24 hours. Then, internal standards were added as described above. The mixture was incubated at 30 °C for 60 min under constant stirring, and then dried under a stream of nitrogen. The residue containing isothiocyanate derivatives (isothiocyanate-mercaptoethanol derivatives) and other hydrolysis compounds were dissolved in 200 µL of acetonitrile/water (1:1) (v/v) and 10 µL of this solution injected onto a Agilent 1100 HPLC system (Agilent). Extracts were separated on a Eclipse XDB-C18 column (150 × 4 mm, particle size 5 μm, Agilent) with a Adsorbosphere C18 all-guard™ cartridge pre-column (Grace, Deerfield, IL). Mobile phase A was water and B methanol. Mobile phase B was 0% at injection, increasing to 10% by 10 min, 100% at 35 min, and held 5 min, then decreased to 0% by 50 min. Flow rates were kept at 0.8 mL/min. The detector wavelength was set at 227 and 271 nm. Response factors for monomeric indolyl derivatives were used from a previous report [40]. Due to a lack of standards for NI3C and NeoASG the standard curve of I3C was applied for quantification of both NI3C and NeoASG. The quantities were expressed as I3C molar equivalent concentrations. 

### Cloning of Broccoli *Epithiospecifier* Modifier *1* (BoESM1)

Using known *Arabidopsis* (NM_112278.2)*, Brassica rapa* (FJ830451.1) and *Brassica napus* (FJ830448.1) gene sequence information, PCR primers were designed with the Primer3 software package (http://frodo.wi.mit.edu/primer3) to isolate the broccoli, cabbage, and cauliflower homologous *ESM1* gene, known to be associated with GS hydrolysis. PCR amplification was performed using the GoTaq® PCR Core System (Promega, Madison, WI, USA) following the protocol described by the manufacturer. The PCR product was separated on 1% TAE gel and purified by using a Qiagen gel extraction kit (QIAGEN, Valencia, CA, USA) according to the manufacturer’s protocols. The amplified PCR products were cloned with pGEM®-T Easy Vector System (Promega), and the clones were sequenced in the W. Carver Biotechnology Center, University of Illinois at Urbana-Champaign. The amino acid sequences deduced from the isolated cDNA sequences were subjected to phylogenetic tree analysis using Clustal W2 (http://www.ebi.ac.uk/Tools/clustalw2/). (Figure S1). Quantitative RT-PCR (qRT-PCR) primers for *BoESM1* were designed based on the consensus sequences of *B. oleracea* (broccoli, cauliflower, and cabbage), *B. napus*, and *B. rapa* cDNA (Table 1).

**Table 1:** List of primers used for qRT-PCR in broccoli.

Target gene (Accession number)	Description	Forward Primer (5’-3’)	Reverse Primer (5’-3’)	Ref
Glucosinolate biosynthesis				
*BoCYP79B2*	*Brassica oleracea* var. *italica* cytochrome P450 (CYP79B2)	AGCCAAGTCCTTCTCAGTCG	ACGAGATAAACCGGAGATCG	[41]
*BoCYP83B1*	*Brassica oleracea* var. *italica* cytochrome P450 (CYP83B1)	ACGGAACCGAGATGAAGAGA	CTCTCTTGAGACGCGCACTA	[41]
*BoCYP79F1*	*Brassica oleracea* var. *italica* cytochrome P450 (CYP79F1)	TCCGATGGTTCTCATGTTGA	AACCGGATATCGCATGTTTC	[41]
*BoCYP83A1*	*Brassica oleracea* var. *italica* cytochrome P450 (CYP83A1)	TCAAGACGCAAGACGTCAAC	CAAGTGGTTCATCCCCATCT	[41]
Glucosinolate hydrolysis				
*BoMyo* (EU004075)	*Brassica oleracea* myrosinase (MYO)	AACGCCTTTCGTTACCCTCT	TCACCTTTCCACCAAATTCC	[41]
*BoESP* (DQ059298)	*Brassica oleracea* var. *italica* epithiospecifier (ESP) protein	CGAGAAGCTCACATGGCATA	CTTGGACGGAGAGATTGACC	[41]
*BoESM1* (FJ830448.1)	*Brassica oleracea* epithiospecifier modifier 1 (ESM1)	ATTCCAAACGGAATCCCGCC	CCGGAGCCCCAAGAATAGAA	
Plant defense				
*BoPR* (EF423806)	*Brassica oleracea* var. *gemmifera* pathogenesis-related (PR) protein	CCACCATTGTTACACCTTGCT	AACCTTTGGGTCAACGAGAA	[41]
Chlorophyll catabolism				
*BoPPH* (OL386R)	*Brassica oleracea pheophytinase*	AGAGGTTATCGGTGAGCCA	GACGAGATGAGGATGGG	[42]
*BoPaO* (*AM388844.1*)	*Brassica oleracea pheophorbide a oxygenase*	GCGAAATTCCCGTCCAGAGTCTC	TTATCTCCGCCGTGCTCTTCTTC	[42]
qRT-PCR controls				
*BoACT1* (AF044573)	*Brassica oleracea* actin (ACT1)	TCTCGATGGAAGAGCTGGTT	GATCCTTACCGAGGGAGGTT	[41]

### RNA Extraction and Quantitative Real Time-PCR

Total RNA was isolated from control and MeJA treated floret tissue samples using the RNeasy Mini Kit (QIAGEN) according to the manufacturer’s instructions. The quantity of RNA was measured using a NanoDrop 3300 spectrophotometer (Thermo Scientific, Waltham, MA). One μg of the total RNA was reverse-transcribed with Superscript™ III First-Strand Synthesis SuperMix for qRT-PCR (Invitrogen, Carlsbad, CA, USA) according to the manufacturer’s instructions. The resulting cDNA samples were diluted to 1/10 their concentrations (v/v) for qRT-PCR. Previously reported primer sets of GS biosynthesis (*BoCYP79B2*, *BoCYP83B1*, *BoCYP79F1*, and *BoCYP83A1*) genes, hydrolysis (*BoMyo*, *BoESP*, and *BoESM1*) genes, a pathogenesis-related (PR) protein (*BoPR*) gene known to be responsive to MeJA, chlorophyll catabolism (*BoPPH* and *BoPaO*) genes, and the broccoli actin gene (*BoACT1*) as a normalization standard were used for qRT-PCR [41,42] (Table 1). Transcript abundance of the broccoli actin (*BoACT1*) gene from each treatment was stable throughout post-harvest storage (Figure S2). The primer sequence sets were synthesized by Integrated DNA Technologies (Coralville, IA, USA). Quantitative Real-time PCR was carried out with the real-time Power SYBR^®^ Green PCR Master Mix (QIAGEN) using Taqman ABI 7900 (Applied Biosystems, Foster city, CA, USA) according to the manufacturer’s instructions. The relative expression ratio was determined with the equation 2-^∆∆Ct^ by normalizing with *BoACT1*, using the Ct values generated by the Taqman ABI 7900 Sequence Detection System Software 2.4 (Applied Biosystems). 

### Statistical Analysis

Statistical analyses were conducted using the JMP 10 software (SAS institute Inc., Cary, NC). Student’s T-tests were used for comparing treatment groups. Fisher’s Least Significant Difference (LSD) test was conducted for comparing treatment group means at *P* ≤ 0.05. Pearson correlation was conducted on all pairs of a GS, hydrolysis product and chlorophyll concentrations, gene expression, and QR inducing activity based on the mean values of each treatment across post-harvest storage dates. The results are presented as means ± SD.

## Results and Discussion

### Ethylene Production and Respiration Rate for Broccoli Florets Subjected to MeJA Treatments

Treatment with 500 µM MeJA significantly increased ethylene production (1.9 fold) in broccoli floret tissues four days after treatment (Figure S3A). Ethylene production dropped significantly during postharvest storage at 4 °C. Ethylene production between control and MeJA treated groups were not significantly different at 10 and 20 days of storage regardless of 1-MCP treatment. There was also no consistent difference in respiration rates among the different treatments at harvest or during postharvest storage (Figure S3B).

### Product Color Measurement for Visual Quality Change and Chlorophyll Concentrations

**Figure 2:**
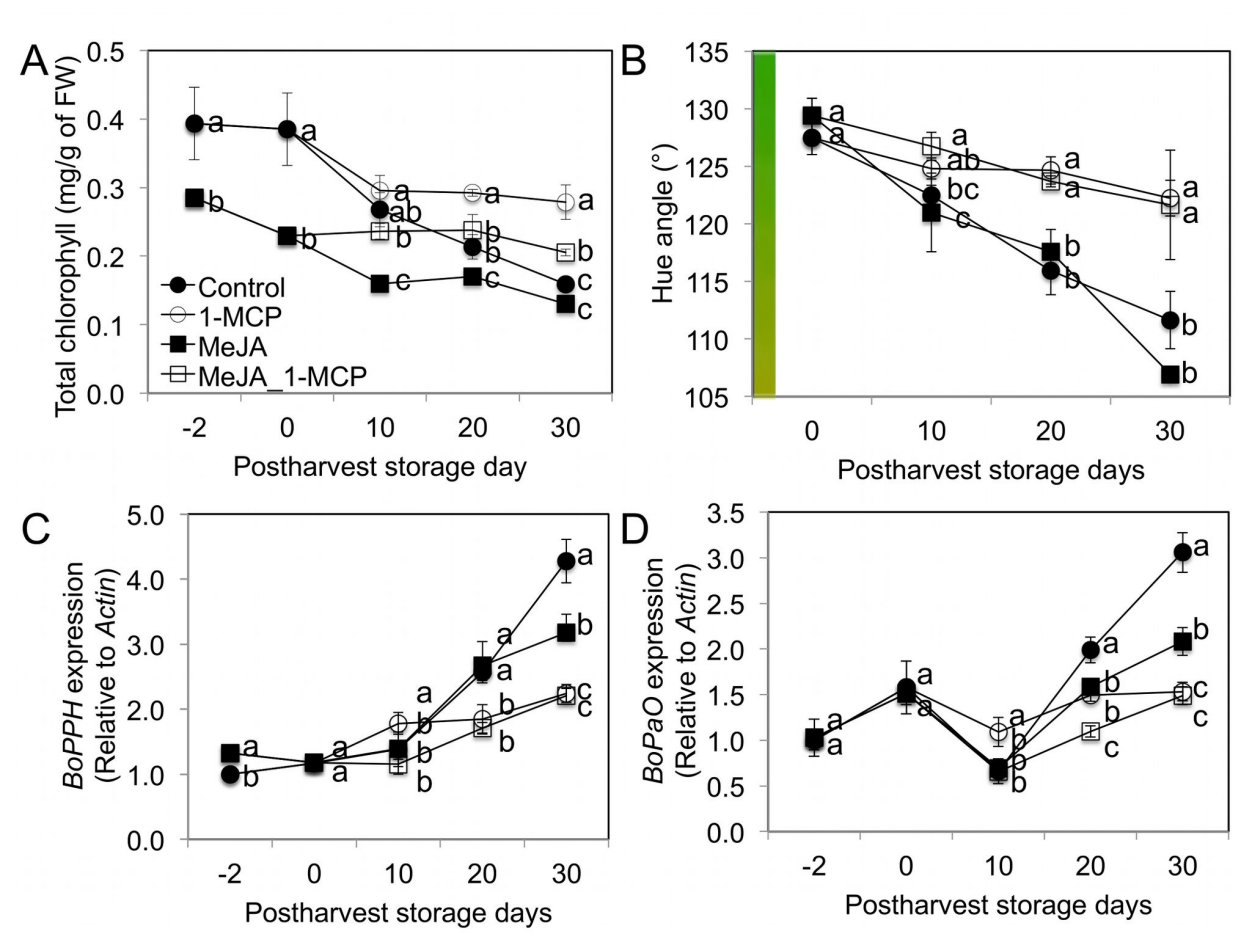
Changes in total chlorophyll content (A), hue angle (B), and gene expression of chlorophyll catabolism genes from pre-harvest MeJA and post-harvest 1-MCP treatments two days prior to harvest, at harvest, and during post-harvest storage at 4 °C. C: broccoli pheophytinase (BoPPH) transcript abundance D: broccoli pheophorbide a oxygenase, (BoPaO) transcript abundance. Different letters indicate significant differences among treatments based on Fisher’s LSD test at P ≤ 0.05. Mean ± SD (n=3).

Images of broccoli florets from the four different treatments from each assay date are presented in Figure 1. There were differences in visual quality between control and 1-MCP treatment groups over the period of post-harvest storage, regardless of MeJA treatment (Figure 1). In order to determine this objectively, tissue chlorophyll concentrations and floret hue angle were measured to quantify visual quality. There was a significant reduction in total chlorophyll content in broccoli florets two (day-2) and four days (head harvest) after MeJA treatment compared to controls (Figure 2A). It was previously reported that MeJA treatment reduced total chlorophyll content in *Arabidopsis thaliana* [43]. Chlorophyll b concentrations are much higher than chlorophyll a in broccoli and showed more dramatic losses during post-harvest storage (data not shown). Hue angle measurements indicated that 1-MCP treatments were associated with superior visual quality throughout the period of post-harvest storage compared to controls and the MeJA treatment (Figure 2B). 

### Chlorophyll Catabolism Gene Expression by Pre-harvest MeJA and Post-harvest 1-MCP Treatments

To investigate the mechanism of chlorophyll and visual quality loss during the post-harvest period, relative transcript abundance of two genes associated with chlorophyll catabolism *BoPPH* and *BoPaO* were assayed by qRT-PCR. Transcript abundance of these genes was significantly greater in the control or MeJA treated broccoli for *BoPPH* at 20 and 30 days of post-harvest storage and for *BoPaO* at 30 days post-harvest storage (Figure 2C, D). There was a significant negative correlation (*r* = -0.642, *P* = 0.007) between *BoPPH* gene expression (Figure 2C) and total chlorophyll concentrations (Figure 2A, Table 2). Hue angle measurements of visual quality were negatively correlated with both *BoPPH* (*r* = -0.868, *P* < 0.001) and *BoPaO* gene expression (*r* = -0.641, *P* = 0.014, Table 2). Down-regulation of expression of these genes to maintain visual quality has been previously reported in broccoli [28]. 1-MCP mediated reduction of ethylene binding to receptor proteins has previously been shown to be responsible for reduced expression of *BoPPH* and *BoPaO*, chlorophyll degradation and associated visual quality loss during post-harvest storage [30].

**Table 2:** Correlations among phytochemicals, QR inducing activity and gene expression of broccoli florets during postharvest storage at 4 °C.

No	Variables	1	2	3	4	5	6	7	8	9	10	11	12	13	14	15	16	17	18	19	20
1	Glucoraphanin	1																			
2	Glucobrassicin	**0.68**																			
3	Gluconasturtiin	0.11	0.39																		
4	4-Methoxyglucobrassicin	**-0.84**	**-0.58**	0.33																	
5	Neoglucobrassicin	-0.05	0.49	**0.88**	0.35																
6	Sulforaphane	-0.42	0.09	**0.71**	**0.70**	**0.86**															
7	PEITC	-0.02	0.21	**0.77**	0.43	**0.84**	**0.71**														
8	I3C	0.34	**0.70**	0.35	-0.21	**0.60**	0.39	0.27													
9	NeoASG	-0.13	0.27	**0.91**	0.46	**0.93**	**0.84**	**0.94**	0.37												
10	NI3C	-0.23	0.21	**0.85**	0.51	**0.91**	**0.86**	**0.89**	0.33	**0.98**											
11	QR inducing activity	-0.49	-0.01	**0.71**	**0.72**	**0.80**	**0.87**	**0.71**	0.21	**0.87**	**0.90**										
12	Total chlorophylls	**0.59**	0.40	-0.49	**-0.81**	-0.43	**-0.60**	**-0.56**	0.10	-0.50	-0.46	**-0.60**									
13	*BoPAO*	0.00	-0.25	-0.02	0.17	-0.24	-0.11	-0.10	-0.27	-0.27	-0.30	-0.36	-0.37								
14	*BoPPH*	-0.27	**-0.58**	0.06	0.47	-0.18	0.06	0.09	-0.39	-0.09	-0.11	-0.10	**-0.64**	**0.87**							
15	*BoCYP79B2*	0.25	0.51	0.03	-0.29	0.28	0.08	-0.02	**0.68**	0.01	0.00	-0.09	0.16	-0.10	-0.20						
16	*BoCYP83B1*	0.23	**0.62**	-0.10	-0.37	0.20	0.04	-0.22	**0.67**	-0.12	-0.11	-0.20	0.32	-0.06	-0.33	**0.88**					
17	*BoCYP83A1*	0.26	0.43	-0.23	-0.40	0.07	-0.08	-0.25	**0.69**	-0.15	-0.12	-0.24	0.46	-0.23	-0.37	**0.81**	**0.81**				
18	*BoCYP79F1*	0.20	0.28	-0.36	-0.40	-0.09	-0.16	-0.36	**0.56**	-0.24	-0.19	-0.30	**0.56**	-0.24	-0.38	**0.58**	**0.65**	**0.94**			
19	Ethylene production	0.44	**0.71**	0.25	-0.31	0.29	0.15	0.02	**0.61**	0.04	-0.02	-0.14	0.15	0.22	-0.15	**0.84**	**0.73**	**0.86**	**0.76**		
20	Hue angle	**0.60**	**0.75**	0.12	**-0.70**	0.16	-0.18	-0.08	0.32	0.11	0.05	-0.07	**0.72**	**-0.64**	**-0.87**	0.18	0.28	0.43	0.49	-0.18	
21	*BoPR*	0.23	0.41	-0.12	-0.25	-0.02	0.39	-0.24	-0.31	-0.11	-0.14	-0.35	0.34	0.38	0.00	**0.80**	**0.85**	**0.67**	**0.61**	**0.69**	0.00

Pearson’s correlation coefficients and *P*-values were calculated based on the means of each treatment over the duration of postharvest storage sampling (n=16, except for ethylene production and hue angle: n=14). Significant positive or negative correlations are highlighted expressed in bold font with dark or light shading, respectively based on *P* ≤ 0.05.

### Pre-harvest MeJA and Post-harvest 1-MCP Treatments Influence GS and GS Hydrolysis Product Concentrations

**Figure 3:**
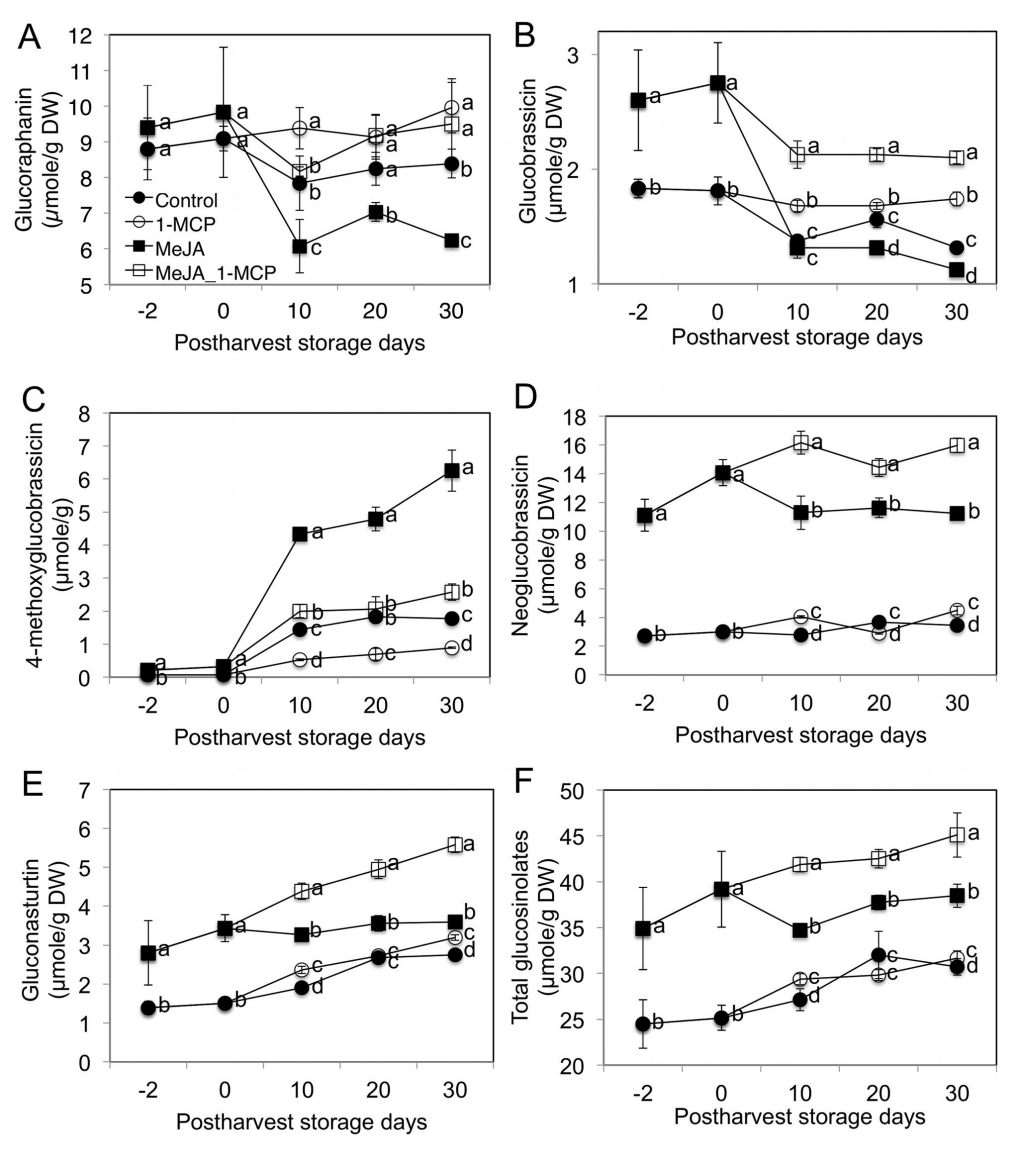
Effect of pre-harvest MeJA and post-harvest 1-MCP treatments on glucosinolate concentrations of florets at 2 days prior to harvest, at harvest, and during post-harvest storage at 4 °C. A: glucoraphanin; B: glucobrassicin; C: 4-methoxyglucobrassicin; D: neoglucobrassicin; E: gluconasturtiin; and F: total glucosinolates. Data are means ± SD (n=3). Different letters indicate significant differences among treatments based on Fisher’s LSD test at P ≤ 0.05.

Treatment with MeJA significantly increased glucobrassicin, 4-methoxyglucobrassicin, neoglucobrassicin, gluconasturtiin, and total glucosinolate concentrations in broccoli floret samples. At harvest (day 0), the relative increase of these GS was 1.58, 4.75, 4.71, 2.28, and 1.49 fold over controls, respectively (Figure 3). In the case of 4-methoxyglucobrassicin, there was more dramatic increase in MeJA treated broccoli compared to control during post-harvest storage. Previous reports indicate MeJA treatments increased glucoraphanin, glucobrassicin, and neoglucobrassicin concentrations in cauliflower curds [20]. Both jasmonate (JA) and MeJA induced significant increases (up to 20-fold) in the concentration of specific indolyl GS in *Brassica napus* (primarily the GS, glucobrassicin), in *B. rapa* (primarily 4-hydroxy glucobrassicin), and in *B. juncea* (both increased) [44]. The different responses to MeJA treatment suggest that variation in GS response to MeJA is species specific. Postharvest 1-MCP treatment maintained glucoraphanin (Figure 3A) and glucobrassicin (Figure 3B) concentrations regardless of MeJA treatment. The combination of MeJA and 1-MCP treatment showed the highest concentrations for glucobrassicin, neoglucobrassicin, gluconasturtiin, and total glucosinolate during post-harvest storage (Figure 3). MeJA treated broccoli showed significant reduction in glucoraphanin, glucobrassicin, and neoglucobrassicin concentrations during the first 10 days of post-harvest storage (Figure 3A, B, and D). This implies that wounding or damage to broccoli heads during post-harvest storage should be minimized to maintain concentrations of glucosinolates associated with health promotion.

The glucobrassicin concentrations of all four treatment groups observed rapidly decreased (Figure 3B) over the duration of post-harvest storage compared to neoglucobrassicin (Figure 3D), which has a structure similar to glucobrassicin (methoxylated glucobrassicin). The concentration of 4-methoxyglucobrassicin was increased in all four treatment groups during post-harvest storage with the MeJA treatment showing the largest increment (Figure 3C). Neoglucobrassicin and 4-methoxyglucobrassicin are products of the glucobrassicin biosynthesis pathway following hydroxylation then methylation, respectively [10]. Considering this biosynthetic pathway, reduction of glucobrassicin may be associated with 4-methoxylation of glucobrassicin. We found that there was a significant correlation between the loss of glucobrassicin and increases of 4-methoxyglucobrassicin (*r* = -0.578, *P* = 0.019, Table 2), implying active GS conversion [13] during post-harvest storage at 4° C.

MeJA treatments were also found to significantly increase gluconasturtiin concentrations at harvest (Figure 3E). Concentrations of gluconasturtiin were observed to increase during the period of post-harvest storage in all the treatments except for the MeJA treated group. Since indolyl GS (glucobrassicin, 4-methoxyglucobrassicin, and neoglucobrassicin) and aromatic GS (gluconasturtiin) share the same biosynthetic pathway, increases in specific GS concentrations may reduce other GS levels with shared precursors. The increment of 4-methoxyglucobrassicin in MeJA treated broccoli florets may interfere with neoglucobrassicin and gluconasturtiin biosynthesis. Post-harvest 1-MCP treatment may contribute to the accumulation of gluconasturtiin by delaying senescence but the effect was greater in MeJA treated broccoli compared to non-MeJA treated broccoli. 

**Figure 4:**
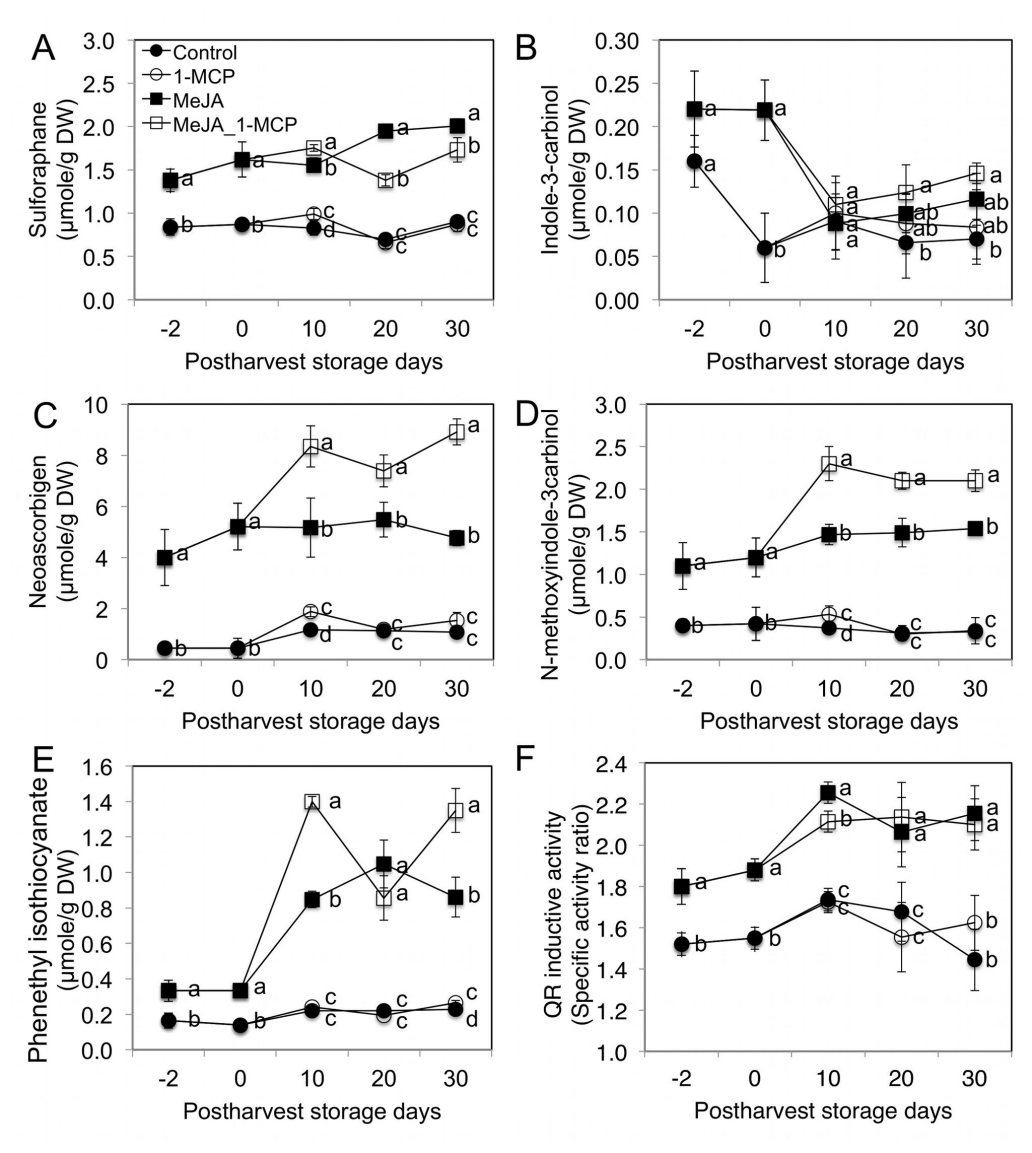
Effect of pre-harvest MeJA and post-harvest 1-MCP treatments on GS hydrolysis product concentrations and QR activity of floret extracts at two days prior to harvest, at harvest, and during post-harvest storage at 4 °C. A: sulforaphane; B: indole-3-carbinol; C: neoascorbigen; D: N-methoxyindole-3-carbinol; E: phenethyl isothiocyanate; and F: QR inducing activity. Data are means ± SD (n=3). Different letters indicate significant differences among treatments based on Fisher’s LSD test at P ≤ 0.05. ^Z^I3C molar equivalent concentration (µmol/g DW).

**Figure 5:**
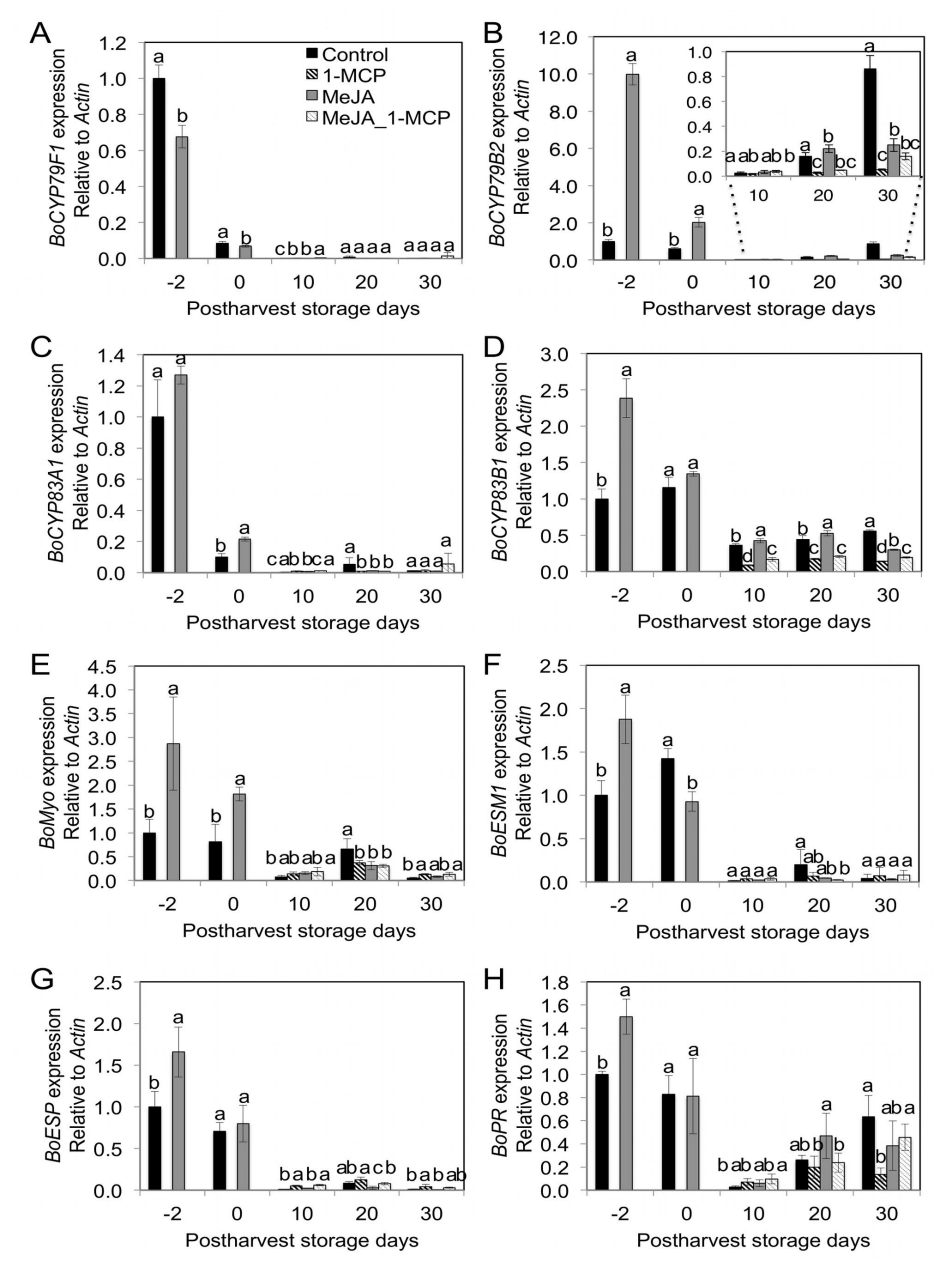
Effect of pre-harvest MeJA and post-harvest 1-MCP treatment on gene expression of GS biosynthetic, hydrolytic, and *PR* genes in broccoli florets two days prior to harvest, at harvest, and during postharvest storage at 4 °C. Different letters indicate significant differences among treatments based on Fisher’s LSD test at P ≤ 0.05. Mean ± SD (n=3).

Interestingly, SF formation was significantly increased by MeJA treatment even though there were no significant increases in glucoraphanin concentrations (Figure 3 and Figure 4). This can be explained by the significantly increased levels in gene expression of myrosinase (*BoMyo*) and *BoESM1* compared to the *BoESP* gene (Figure 5). Myrosinase activity was significantly (58%) increased by MeJA treatment at four days after treatment (Figure S4). MeJA treatment not only increased gluconasturtiin concentration but also PEITC formation (Figure 3 and Figure S5). NeoASG concentrations were significantly increased by MeJA treatment and maintained elevated concentrations during post-harvest storage (Figure 4). The major hydrolysis product of neoglucobrassicin was NeoASG (Figure 4C, D). I3C concentrations were observed to be significantly increased in only the MeJA treatments at harvest (Figure 4B). 

### GS Biosynthetic, Hydrolytic, and Pathogenesis Related-Protein (PR) Gene Expression Changes Mediated by MeJA and/or 1-MCP treatments

The amino acid sequences deduced from the isolated *B. oleracea ESM1* gene sequences corresponded to the *B. napus* (96%), *B. rapa* (95%) and *Arabidopsis thaliana* protein, ESM1 (79%), respectively, which suggests similar gene product function in broccoli by sharing homologous protein motifs (Figure S1). In order to evaluate the effects of MeJA and 1-MCP treatments at harvest and during post-harvest storage, gene expression of GS biosynthetic (*BoCYP79F1, BoCYP79B2*, *BoCYP83A1*, and *BoCYP83B1*), hydrolytic (*BoMyo, BoESP*, and *BoESM1*), and pathogenesis related (*BoPR*) genes were measured by qRT-PCR. While gene expression of *BoCYP79F1* (0.7 fold) was significantly decreased by MeJA treatment compared to control at two days after treatment, transcript abundance of *BoCYP79B2* (10.0 fold), *BoCYP83A1* (1.3 fold, non-significant increase)*, BoCYP83B1* (2.4 fold), *BoMyo* (2.9 fold)*, BoESM1* (1.9 fold), *BoESP* (1.7 fold), and *BoPR* (1.5 fold) were non-significantly or significantly increased compared to controls at two days after treatment (Figure 5). The elevated transcript abundance observed at 2 days after MeJA treatment was dramatically reduced with post-harvest storage at 4 °C (Figure 5). While gene expression levels during post-harvest storage were low and not dramatically different among treatments for many of the genes, transcript abundance for *BoCYP79B2*, *BoCYP83B1*, and *BoPR* gradually increased over the duration of storage. The mRNA expression levels of these genes may explain the increase in indolyl GS during post-harvest storage as was previously reported [45]. Decreased gene expression of *BoCYP79B2* and *BoCYP83B1* associated with 1-MCP treatment is in agreement with previous research in *Arabidopsis* showing that that these GS biosynthetic genes are stimulated by elevated MeJA-mediated ethylene production [11,46,47]. 

### Correlation between Gene Expression and GS Concentrations

There were significant correlations between glucobrassicin concentrations and *BoCYP83B1* (*r* = 0.622, *P* = 0.010) expression, genes that are involved in up-stream biosynthesis of indolyl and aromatic GS (Table 2). *BoCYP79B2* (*r* = 0.841, *P* < 0.001), *BoCYP83B1* (*r* = 0.732, *P* = 0.001), *BoCYP83A1* (*r* = 0.863, *P* < 0.001), and *BoCYP79F1* (*r* = 0.762, *P* < 0.001) gene expression also correlated with ethylene production indicating that GS biosynthesis is an ethylene-mediated response as described above [11,46,47] (Table 2). In addition, *BoPR* gene expression was significantly correlated with ethylene production (*r* = 0.694, *P* = 0.006) and expressions of four GS biosynthesis genes above in our samples (Table 2). *BoPR* expression is modulated by the salicylic acid (SA), MeJA, and ethylene signaling pathways [11]. 

The observed reductions in glucobrassicin with complimentary increases in 4-methoxyglucobrassicin concentrations are likely a result of the 4-methoxylation of glucobrassicin. The *Arabidopsis CYP81F2* gene is involved in accumulation of 4-methoxyglucobrassicin synthesized from glucobrassicin in response to pathogen infection [13,14]. The loss of glucobrassicin in MeJA treated broccoli during the post-harvest storage is possibly due to the higher levels of *BoCYP81F* subfamily transcripts compared to *BoCYP79B2* and *BoCYP83B1* gene expression. Mikkelsen et al. (2003) [11] reported that enzyme activities responsible for the *N*-methoxylation of glucobrassicin are strongly induced by MeJA treatment and this induction is suppressed by ACC. Recently, it has been shown that the *CYP81F4* gene product is involved in N-hydroxylation of glucobrassicin to synthesize neoglucobrassicin [13]. As Mikkelsen et al. (2003) [11] observed, it has been also reported that *CYP81F4* was up-regulated by MeJA and down-regulated by ethylene in *Arabidopsis* (5.81 fold) [48,49]. Consequently, ethylene accumulation in MeJA treated broccoli favors the pathway of methoxylation from glucobrassicin to 4-methoxyglucobrassicin rather than formation of neoglucobrassicin during post-harvest storage. Favoring the methoxylation pathway by accumulation of ethylene or the ethylene precursor, ACC would facilitate defense against postharvest pathogens since 4-methoxyglucobrassicin has been shown to be antibiotic to fungi [14,50]. PEITC, the hydrolysis product of gluconasturtiin has also been reported to possess antifungal activity [51]. The increased levels of gluconasturtiin may be associated with up-regulation of *BoCYP83B1*, which is involved in both indolyl and aromatic GS biosynthesis. Enhanced levels of PEITC during the post-harvest storage could also be associated with antifungal defense.

### Correlations between QR inducing Activity of Broccoli and Enhanced GS and Hydrolysis Products

QR inducing activity of MeJA treated broccoli floret extracts was significantly increased compared to controls at 2 and 4 days after MeJA treatment and throughout the course of post-harvest storage (Figure 4F). In order to elucidate the major QR inducers in MeJA treated broccoli, correlation analysis was conducted between QR inducing activity and other variables (Table 2). Significant positive correlations were observed between QR inducing activity and the GS concentrations of gluconasturtiin (*r* = 0.712, *P* = 0.004), 4-methoxyglucobrassicin (*r* = 0.716, *P* = 0.002), and neoglucobrassicin (*r* = 0.804, *P* < 0.001) in broccoli floret sample extracts during post-harvest storage at 4 °C (Table 2). Previously, 4-methoxyindole-3-carbinol has been reported to provide *in vitro* antiproliferation activity in two different human colon cancer cells lines [52].

 Hydrolysis products of GS, SF (*r* = 0.864, *P* < 0.001), PEITC (*r* = 0.713, *P* = 0.004), NeoASG (*r* = 0.874, *P* < 0.001), and NI3C (*r* = 0.899, *P* < 0.001) were also correlated with QR activity (Table 2). The increased QR activity may be due to the increased concentrations of SF, PEITC, and/or hydrolysis products of neoglucobrassicin including NI3C and NeoASG. SF and PEITC formation were significantly increased by MeJA treatment (Figure S4). 

 This increased isothiocyanate formation induced by MeJA treatment also suggests that plant defense against herbivores may be involved in enhancing isothiocyanate formation as previous research reported that increased allyl isothiocyanate concentrations reduced survival and growth, and increased development time of *Pieris rapae* [53]. The detailed mechanism of how isothiocyanate formation is increased by MeJA treatment or insect invasion is not yet fully understood. However, a possible explanation could be related to the proteins and/or co-factors associated with hydrolysis. MeJA treatment increased gene expression of broccoli myrosinase as previous research reported [54]. The protein co-factor ESP when bound to myrosinase favors hydrolysis and conversion of GS products into nitriles [17] while elevated levels of EMS1 protein in *Arabidopsis* has been associated with enhanced hydrolysis of GS into isothiocyanate products [18]. Compared to broccoli myrosinase, transcript abundance of *BoESP* was not dramatically increased. Increased levels of unbound myrosinase, free of the co-factor, ESP favors the generation of isothiocyanates instead of QR inactive nitrile forms [17]. If co-factors such as ESP and ESM1 act competitively to bind with myrosinase, an increase of *ESM1* gene expression should lead to enhanced isothiocyanate formation as was observed [55]. Compared to gene expression of myrosinase and its co-factors at two days after MeJA treatment, transcript abundance decreased at four days after treatment. This indicates how rapidly the regulatory response of herbivore defense mechanisms can act. Since isothiocyanates are toxic to both plants and herbivores*, Brassica* plants will also produce enhanced levels of detoxifying enzymes such as glutathione transferase for isothiocyanate neutralization [56]. To minimize metabolic costs under non-invasive conditions and reduce isothiocyanate autotoxicity the plant can lower glucosinolates and myrosinase biosynthesis to allocate resources for growth and development while maintaining the capacity to rapidly respond to biotic stress by selectively modifying expression of GS biosynthetic genes and *BoMyo* and its associated protein co-factors.

**Figure 6:**
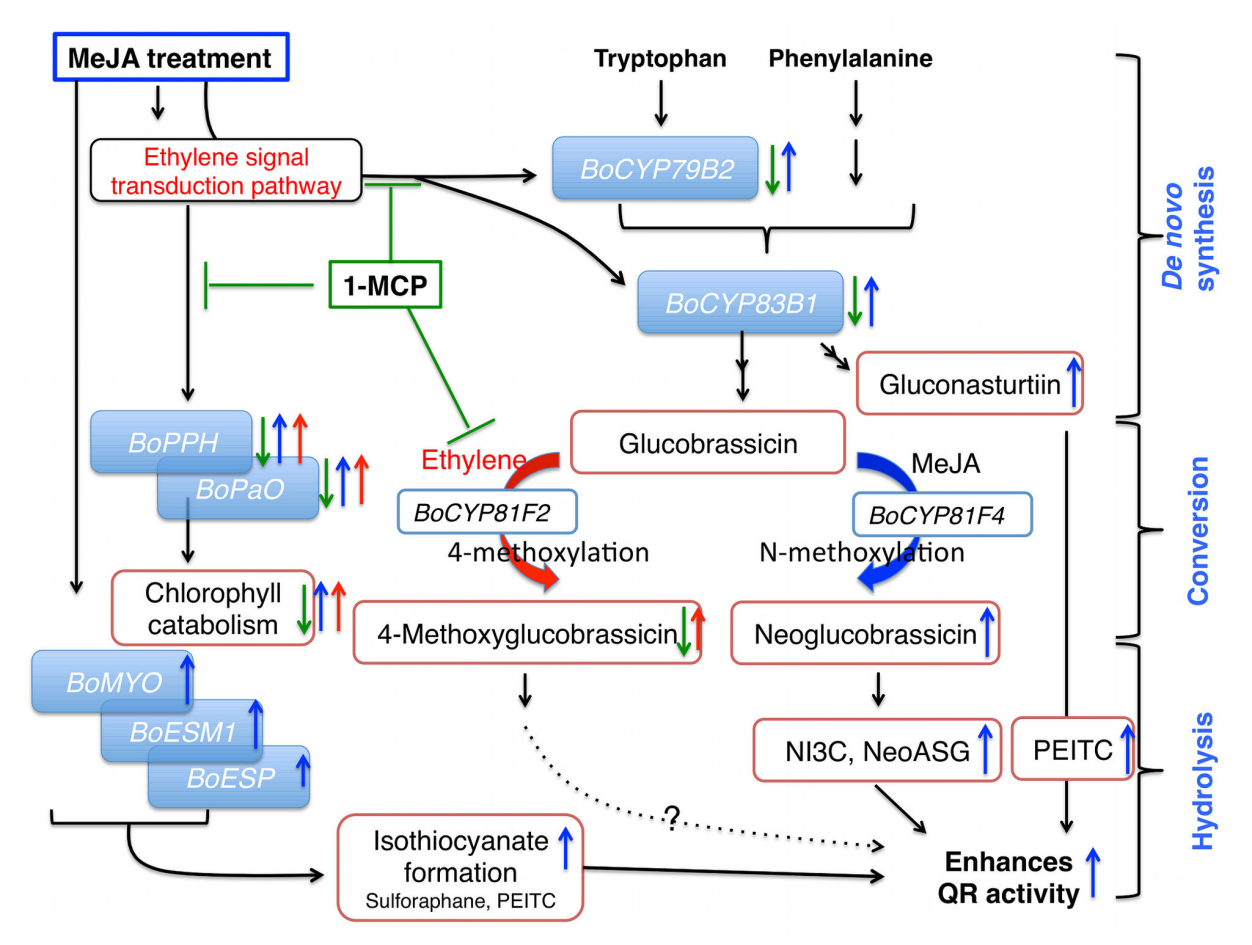
Proposed model of pre-harvest MeJA and post-harvest 1-MCP treatment effects on GS biosynthesis, hydrolysis, QR bioactivity, and visual quality of broccoli florets during post-harvest storage at 4 °C. Pre-harvest MeJA increases indolyl and aromatic GS biosynthesis (*de* *novo* GS biosynthesis). Ethylene accumulation induces 4-methoxylation of glucobrassicin rather than *N*-methoxylation of glucobrassicin during post-harvest (conversion) but 1-MCP maintains glucobrassicin concentrations and reduces indolyl GS biosynthesis during post-harvest by inhibiting ethylene mediated GS biosynthesis. MeJA enhances synthesis of myrosinase and the hydrolysis of GS to favor isothiocyanate formation in the case of glucoraphanin and gluconasturtiin by modulating *BoESM1* and *BoESP* (hydrolysis). 1-MCP treatment maintained postharvest quality by reducing chlorophyll catabolism gene expression of *BoPPH* and *BoPaO*. This study demonstrates the combined treatment of MeJA and 1-MCP increased QR activity without post-harvest quality loss. Blue arrows describe MeJA regulated gene expression; green arrows 1-MCP regulated gene expression; and red arrows ethylene regulated gene expression.

A standard format for comparing the efficacy of elicitors of QR induction is determining concentrations required for a two-fold increase in activity (CD value). The CD value of NI3C, NeoASG, PEITC and SF are 35, 38.5, 5.0, and 0.2 µM, respectively [3]. Even though SF concentrations observed in our study are smaller than those of NeoASG (Figure 4), the relative SF bioactivity suggests it is the major contributor toward enhanced QR inducing activity in our extracts, although MeJA-mediated increases in other GS and their hydrolysis products are also likely contributors. More research is needed to determine if the hydrolysis products of 4-methoxyglucobrassicin induce QR activity or not (Figure 6). 

### Proposed Model of Pre-harvest MeJA and Post-harvest 1-MCP Treatment Effects on Postharvest Physiology and Health Promoting Bioactivity of Broccoli Florets

Our study suggests that pre-harvest MeJA and postharvest 1-MCP treatments have influence on GS *de novo* synthesis, conversion, and hydrolysis (Figure 6). In most previous studies typically only MeJA mediated GS concentration changes were reported without information about the hydrolysis products, which are the active agents of QR induction. This present study suggests that MeJA treatment not only increases concentrations of certain GS (Figure 3), but can also increase sulforaphane and PEITC conversion rates (Figure S5) from precursor GS by modulating synthesis of myrosinase protein and its cofactors including *BoESP* and *BoEMS1* (Figure 5). This study confirmed that *de novo* GS biosynthesis in broccoli florets occurs during post-harvest storage. As Figure 5B and D show, *BoCYP79B2* and *BoCYP83B1* are responsible for the *de novo* indolyl and aromatic GS biosynthesis during post-harvest storage. GS biosynthesis genes are regulated by ethylene-mediated signals. Thus, 1-MCP treatment reduced gene expression of GS biosynthesis genes including *BoCYP79B2* and *BoCYP83B1* by blocking ethylene and ethylene receptor protein binding during postharvest storage (Figure 5B and D). Ethylene accumulation induces 4-methoxylation of glucobrassicin rather than *N*-methoxylation of glucobrassicin during post-harvest but 1-MCP treatment helped to maintain glucobrassicin and neoglucobrassicin concentrations by inhibiting 4-methoxylation. 1-MCP treatment maintained postharvest quality (Figure 1) by reducing chlorophyll catabolism gene expressions including *BoPPH* and *BoPaO* (Figure 2). This study demonstrates the combined treatment of MeJA and 1-MCP can improve the delivery of anti-cancer compounds to consumers while maintaining postharvest visual quality (Figure 6). 

## Supporting Information

Figure S1 **Phylogenetic tree of *epithiospecifier* modifier *1* (ESM1) co-factor associated with glucosinolate hydrolysis based on the amino acid sequences deduced from the isolated cDNA sequences.** *Brassica oleracea* consensus (cabbage, broccoli, and cauliflower), *Brassica rapa* *ssp.* *perkinesis* (ACO57702.1), *Brassica napus* (ACO57703.1), and *Arabidopsis thaliana* *ESM1* (ABB90255.1) used to construct phylogenetic tree. The values in parenthesis are amino acid sequence similarity with *B. oleracea* consensus by using NCBI BLAST search. The tree was constructed using Clustal W2 (http://www.ebi.ac.uk/Tools/clustalw2/).
(TIF)

Figure S2 **Transcript abundance of *BoACT1* at two days before harvest, at harvest, and during post-harvest storage at 4 °C.**
(TIF)

Figure S3 **Effects of pre-harvest MeJA and post-harvest 1-MCP treatments on ethylene production and respiration rate of broccoli florets at harvest and at 10, 20, and 30 days of post-harvest storage at 4 °C.** Different letters indicate significant differences among treatments based on Fisher’s LSD test at P ≤ 0.05. Mean ± SD (n=3).
(TIF)

Figure S4 **Effect of MeJA treatment on broccoli floret myrosinase activity at harvest.** Student’s T-test was conducted to determine significance. Mean ± SD (n=3).
(TIF)

Figure S5 **Sulforaphane and phenethyl isothiocyanate (PEITC) conversion from glucoraphanin and gluconasturtiin at two days before harvest, at harvest, and during post-harvest storage at 4 °C.** Different letters indicate significant differences among treatments based on Fisher’s LSD test at P ≤ 0.05. Mean ± SD (n=3).
(TIF)

## References

[1] van Poppel G, Verhoeven DT, Verhagen H, Goldbohm RA (1999) *Brassica* vegetables and cancer prevention. Epidemiology and mechanisms. Adv Exp Med Biol 472: 159-168. doi:10.1007/978-1-4757-3230-6_14. PubMed: 10736624. 10736624

[2] Wallig MA, Kingston S, Staack R, Jefferey EH (1998) Induction of rat pancreatic glutathione S-transferase and quinone reductase activities by a mixture of glucosinolate breakdown derivatives found in Brussels sprouts. Food Chem Toxicol 36: 365-373. doi:10.1016/S0278-6915(97)00156-7. PubMed: 9662411. 9662411

[3] Zhang Y, Talalay P, Cho CG, Posner GH (1992) A major inducer of anticarcinogenic protective enzymes from broccoli: isolation and elucidation of structure. Proc Natl Acad Sci U S A 89: 2399-2403. doi:10.1073/pnas.89.6.2399. PubMed: 1549603. 1549603 PMC48665

[4] Hwang ES, Jeffery EH (2003) Evaluation of urinary N-acetyl cysteinyl allyl isothiocyanate as a biomarker for intake and bioactivity of Brussels sprouts. Food Chem Toxicol 41: 1817-1825. doi:10.1016/S0278-6915(03)00235-7. PubMed: 14563407. 14563407

[5] Cuendet M, Oteham CP, Moon RC, Pezzuto JM (2006) Quinone reductase induction as a biomarker for cancer chemoprevention. J Nat Prod 69: 460-463. doi:10.1021/np050362q. PubMed: 16562858. 16562858 PMC1876771

[6] Kim DJ, Shin DH, Ahn B, Kang JS, Nam KT et al. (2003) Chemoprevention of colon cancer by Korean food plant components. Mutat Res 523-524: 99-107. doi:10.1016/S0027-5107(02)00325-1. PubMed: 12628507. 12628507

[7] Neave AS, Sarup SM, Seidelin M, Duus F, Vang O (2005) Characterization of the *N*-methoxyindole-3-carbinol (NI3C) induced cell cycle arrest in human colon cancer cell lines. Toxicol Sci 83: 126-135 10.1093/toxsci/kfi008 15483186

[8] Jump SM, Kung J, Staub R, Kinseth MA, Cram EJ et al. (2008) *N*-Alkoxy derivatization of indole-3-carbinol increases the efficacy of the G1 cell cycle arrest and of I3C-specific regulation of cell cycle gene transcription and activity in human breast cancer cells. Biochem Pharmacol 75: 713-724. doi:10.1016/j.bcp.2007.09.024. PubMed: 18023427. 18023427 PMC3422660

[9] Bak Nielsen H Sr, Halkier B (1998) The presence of CYP79 homologues in glucosinolate-producing plants shows evolutionary conservation of the enzymes in the conversion of amino acid to aldoxime in the biosynthesis of cyanogenic glucosides and glucosinolates. Plant Mol Biol 38: 725-734. doi:10.1023/A:1006064202774. PubMed: 9862490. 9862490

[10] Sønderby IE, Geu-Flores F, Halkier BA (2010) Biosynthesis of glucosinolates - gene discovery and beyond. Trends Plant Sci 15: 283-290. doi:10.1016/j.tplants.2010.02.005. PubMed: 20303821. 20303821

[11] Mikkelsen MD, Petersen BL, Glawischnig E, Jensen AB, Andreasson E et al. (2003) Modulation of CYP79 genes and glucosinolate profiles in *Arabidopsis* by defense signaling pathways. Plant Physiol 131: 298-308. doi:10.1104/pp.011015. PubMed: 12529537. 12529537 PMC166809

[12] Hopkins RJ, van Dam NM, van Loon JJ (2009) Role of glucosinolates in insect-plant relationships and multitrophic interactions. Annu Rev Entomol 54: 57-83. doi:10.1146/annurev.ento.54.110807.090623. PubMed: 18811249. 18811249

[13] Pfalz M, Mikkelsen MD, Bednarek P, Olsen CE, Halkier BA et al. (2011) Metabolic engineering in *Nicotiana* *benthamiana* reveals key enzyme functions in *Arabidopsis* indole glucosinolate modification. Plant Cell 23: 716-729. doi:10.1105/tpc.110.081711. PubMed: 21317374. 21317374 PMC3077789

[14] Bednarek P, Pislewska-Bednarek M, Svatos A, Schneider B, Doubsky J et al. (2009) A glucosinolate metabolism pathway in living plant cells mediates broad-spectrum antifungal defense. Science 323: 101-106. doi:10.1126/science.1163732. PubMed: 19095900. 19095900

[15] Kim JH, Lee BW, Schroeder FC, Jander G (2008) Identification of indole glucosinolate breakdown products with antifeedant effects on *Myzus* *persicae* (green peach aphid). Plant J 54: 1015-1026. doi:10.1111/j.1365-313X.2008.03476.x. PubMed: 18346197. 18346197

[16] Bones AM, Rossiter JT (1996) The myrosinase-glucosinolate system, its organisation and biochemistry. Physiol Plant 97: 194-208. doi:10.1111/j.1399-3054.1996.tb00497.x.

[17] Matusheski NV, Swarup R, Juvik JA, Mithen R, Bennett M et al. (2006) Epithiospecifier protein from broccoli (*Brassica* *oleracea* L. *ssp.* *italica*) inhibits formation of the anticancer agent sulforaphane. J Agric Food Chem 54: 2069-2076. doi:10.1021/jf0525277. PubMed: 16536577. 16536577

[18] Zhang Z, Ober JA, Kliebenstein DJ (2006) The gene controlling the quantitative trait locus EPITHIOSPECIFIER MODIFIER1 alters glucosinolate hydrolysis and insect resistance in *Arabidopsis* . Plant Cell 18: 1524-1536. doi:10.1105/tpc.105.039602. PubMed: 16679459. 16679459 PMC1475484

[19] Howe GA, Jander G (2008) Plant immunity to insect herbivores. Annu Rev Plant Biol 59: 41-66. doi:10.1146/annurev.arplant.59.032607.092825. PubMed: 18031220. 18031220

[20] Ku KM, Choi J-H, Kushad MM, Jeffery EH, Juvik JA (2013) Pre-harvest methyl jasmonate treatment enhances cauliflower chemoprotective attributes without a loss in postharvest quality. Plant Foods Hum Nutr 68: 113-117. doi:10.1007/s11130-013-0356-y. PubMed: 23640295. 23640295

[21] Watanabe K, Kamo T, Nishikawa F, Hyodo H (2000) Effect of methyl jasmonate on senescence of broccoli florets. Engei Gakkai Zasshi 69: 605-610. doi:10.2503/jjshs.69.605.

[22] Paliyath G (2008) Postharvest biology and technology of fruits, vegetables, and flowers. xii. Ames, IA: Wiley-Blackwell. 482 pp.

[23] Ku VVV, Wills RBH (1999) Effect of 1-methylcyclopropene on the storage life of broccoli. Postharvest Biol Technol 17: 127-132. doi:10.1016/S0925-5214(99)00042-3.

[24] Gaofeng Y, Bo S, Jing Y, Qiaomei W (2010) Effect of 1-methylcyclopropene on shelf life, visual quality, antioxidant enzymes and health-promoting compounds in broccoli florets. Food Chem 118: 774-781. doi:10.1016/j.foodchem.2009.05.062.

[25] Ma G, Zhang L, Kato M, Yamawaki K, Asai T et al. (2010) Effect of 1-methylcyclopropene on the expression of genes for ascorbate metabolism in postharvest broccoli. Postharvest Biol Technol 58: 121-128. doi:10.1016/j.postharvbio.2010.05.011.

[26] Gang M, Ran W, Cheng-Rong W, Kato M, Yamawaki K et al. (2009) Effect of 1-methylcyclopropene on expression of genes for ethylene biosynthesis enzymes and ethylene receptors in post-harvest broccoli. Plant Growth Regul 57: 223-232. doi:10.1007/s10725-008-9339-7.

[27] Dixon GR (2007) Vegetable *Brassicas* and related crucifers: CABI Pub.

[28] Bücherta AM, Civello PM, Martínez GA (2011) Chlorophyllase versus pheophytinase as candidates for chlorophyll dephytilation during senescence of broccoli. J Plant Physiol 168: 337-343. doi:10.1016/j.jplph.2010.07.011. PubMed: 20727617. 20727617

[29] Gomez-Lobato ME, Civello PM, Martínez GA (2012) Effects of ethylene, cytokinin and physical treatments on *BoPaO* gene expression of harvested broccoli. J Sci Food Agric 92: 151-158. doi:10.1002/jsfa.4555. PubMed: 21732385. 21732385

[30] Gomez-Lobato ME, Hasperué JH, Civello PM, Chaves AR, Martínez GA (2012) Effect of 1-MCP on the expression of chlorophyll degrading genes during senescence of broccoli (*Brassica* *oleracea* . p. L). Sci Hortic (Amsterdam) 144: 208-211

[31] Ku KM, Juvik JA (2012) Optimum methyl jasmonate application to enhance glucosinolate concentration in broccoli florets. Hortsci 47: S311.

[32] Arnon DI (1949) Copper enzymes in isolated chloroplasts. Polyphenoloxidase in *Beta* *Vulgaris* . Plant Physiol 24: 1-15. doi:10.1104/pp.24.1.1. PubMed: 16654194. 16654194 PMC437905

[33] Brown AF, Yousef GG, Jeffery EH, Klein BP, Wallig MA et al. (2002) Glucosinolate profiles in broccoli: Variation in levels and implications in breeding for cancer chemoprotection. J Am Soc Hort Sci 127: 807-813.

[34] Peñas E, Frias J, Martínez-Villaluenga C, Vidal-Valverde C (2011) Bioactive compounds, myrosinase activity, and antioxidant capacity of white cabbages grown in different locations of Spain. J Agric Food Chem 59: 3772-3779. doi:10.1021/jf200356m. PubMed: 21413789. 21413789

[35] Pang Q, Chen S, Li L, Yan X (2009) Characterization of glucosinolate-myrosinase system in developing salt cress *Thellungiella* *halophila* . Physiol Plant 136: 1-9. doi:10.1111/j.1399-3054.2009.01211.x. PubMed: 19508363. 19508363

[36] Li X, Kushad MM (2004) Correlation of glucosinolate content to myrosinase activity in horseradish (*Armoracia* *rusticana*). J Agric Food Chem 52: 6950-6955. doi:10.1021/jf0401827. PubMed: 15537302. 15537302

[37] Bergmeyer HU (1974). ethods Enzymatic Anal: 1212.

[38] Prochaska HJ, Santamaria AB (1988) Direct measurement of NAD(P)H:quinone reductase from cells cultured in microtiter wells: a screening assay for anticarcinogenic enzyme inducers. Anal Biochem 169: 328-336. doi:10.1016/0003-2697(88)90292-8. PubMed: 3382006. 3382006

[39] Wilson EA, Ennahar S, Zhao M, Bergaentzle M, Marchioni E et al. (2011) Simultaneous determination of various isothiocyanates by RP-LC following precolumn derivatization with mercaptoethanol. Chromatographia 73: 137-142. doi:10.1007/s10337-010-1878-1. PubMed: 21765536. 21765536 PMC3098979

[40] Agerbirk N, Olsen CE, Sørensen H (1998) Initial and final products, nitriles, and ascorbigens produced in myrosinase-catalyzed hydrolysis of indole glucosinolates. J Agric Food Chem 46: 1563-1571. doi:10.1021/jf9708498.

[41] Kim HS (2011) Functional studies of lignin biosynthesis genes and putative flowering gene in *Miscanthus**×**giganteus* and studies on indolyl glucosinolate biosynthesis and translocation in *Brassica**oleracea* [Dissertation (Ph. D.)]. Urbana-Champaign: University of Illinois at Urbana-Champaign..

[42] Hasperué JH, Gómez-Lobato ME, Chaves AR, Civello PM, Martínez GA (2013) Time of day at harvest affects the expression of chlorophyll degrading genes during postharvest storage of broccoli. Postharvest Biol Technol 82: 22-27. doi:10.1016/j.postharvbio.2013.02.021.

[43] Jung S (2004) Effect of chlorophyll reduction in Arabidopsis thaliana by methyl jasmonate or norflurazon on antioxidant systems. Plant Physiol Biochem 42: 225-231. doi:10.1016/j.plaphy.2004.01.001. PubMed: 15051046. 15051046

[44] Bodnaryk RP (1994) Potent effect of jasmonates on indole glucosinolates in oilseed rape and mustard. Phytochem 35: 301-305. doi:10.1016/S0031-9422(00)94752-6.

[45] Rodrigues AS, Rosa EAS (1999) Effect of post-harvest treatments on the level of glucosinolates in broccoli. J Sci Food Agric 79: 1028-1032. doi:10.1002/(SICI)1097-0010(19990515)79:7.

[46] Stotz HU, Pittendrigh BR, Kroymann J, Weniger K, Fritsche J et al. (2000) Induced plant defense responses against chewing insects. Ethylene signaling reduces resistance of *Arabidopsis* against Egyptian cotton worm but not diamondback moth. Plant Physiol 124: 1007-1018. doi:10.1104/pp.124.3.1007. PubMed: 11080278. 11080278 PMC59200

[47] Chang C, Shockey JA (1999) The ethylene-response pathway: signal perception to gene regulation. Curr Opin Plant Biol 2: 352-358. doi:10.1016/S1369-5266(99)00004-7. PubMed: 10508761. 10508761

[48] Hall BP, Shakeel SN, Amir M, Ul Haq N, Qu X et al. (2012) Histidine kinase activity of the ethylene receptor *ETR1* facilitates the ethylene response in *Arabidopsis* . Plant Physiol 159: 682-695. doi:10.1104/pp.112.196790. PubMed: 22467798. 22467798 PMC3375934

[49] Kai K, Takahashi H, Saga H, Ogawa T, Kanaya S et al. (2011) Metabolomic characterization of the possible involvement of a Cytochrome P450, CYP81F4, in the biosynthesis of indolic glucosinolate in *Arabidopsis* . Plant Biotechnol 28: 379-385. doi:10.5511/plantbiotechnology.11.0704b.

[50] Clay NK, Adio AM, Denoux C, Jander G, Ausubel FM (2009) Glucosinolate metabolites required for an *Arabidopsis* innate immune response. Science 323: 95-101. doi:10.1126/science.1164627. PubMed: 19095898. 19095898 PMC2630859

[51] Drobnica L, Zemanová M, Nemec P, Antos K, Kristián P et al. (1967) Antifungal activity of isothiocyanates and related compounds. I. Naturally occurring isothiocyanates and their analogues. Appl Microbiol 15: 701-709. PubMed: 6049294. 6049294 10.1128/am.15.4.701-709.1967 PMC547041

[52] Kronbak R, Duus F, Vang O (2010) Effect of 4-methoxyindole-3-carbinol on the proliferation of colon cancer cells *in* *vitro*, when treated alone or in combination with indole-3-carbinol. J Agric Food Chem 58: 8453-8459. doi:10.1021/jf101806t. PubMed: 20593832. 20593832

[53] Agrawal AA, Kurashige NS (2003) A role for isothiocyanates in plant resistance against the specialist herbivore *Pieris* *rapae* . J Chem Ecol 29: 1403-1415. doi:10.1023/A:1024265420375. PubMed: 12918924. 12918924

[54] Jun B-K, Seo S-G, Kim J-S, Lee Y, Shin M-R et al. (2011) Molecular cloning and expression analysis of *Bro-GS-elong* and *Bro-myro* from *Brassica* *oleracea* . Genes Genomics 33: 299-305. doi:10.1007/s13258-011-0031-3.

[55] Baskar V, Gururani MA, Yu JW, Park SW (2012) Engineering glucosinolates in plants: Current knowledge and potential uses. Appl Biochem Biotechnol 168: 1694-1717. doi:10.1007/s12010-012-9890-6. PubMed: 22983743. 22983743

[56] Sellam A, Poupard P, Simoneau P (2006) Molecular cloning of *AbGst1* encoding a glutathione transferase differentially expressed during exposure of *Alternaria* *brassicicola* to isothiocyanates. FEMS Microbiol Lett 258: 241-249. doi:10.1111/j.1574-6968.2006.00223.x. PubMed: 16640580. 16640580

