# Supplementary figures and images for: Methyl Jasmonate and 1-Methylcyclopropene Treatment Effects on Quinone Reductase Inducing Activity and Post-Harvest Quality of Broccoli
Source: PLoS One. 2013 Oct 16;8(10):e77127. doi: 10.1371/journal.pone.0077127 (PMC3797761; doi:10.1371/journal.pone.0077127)

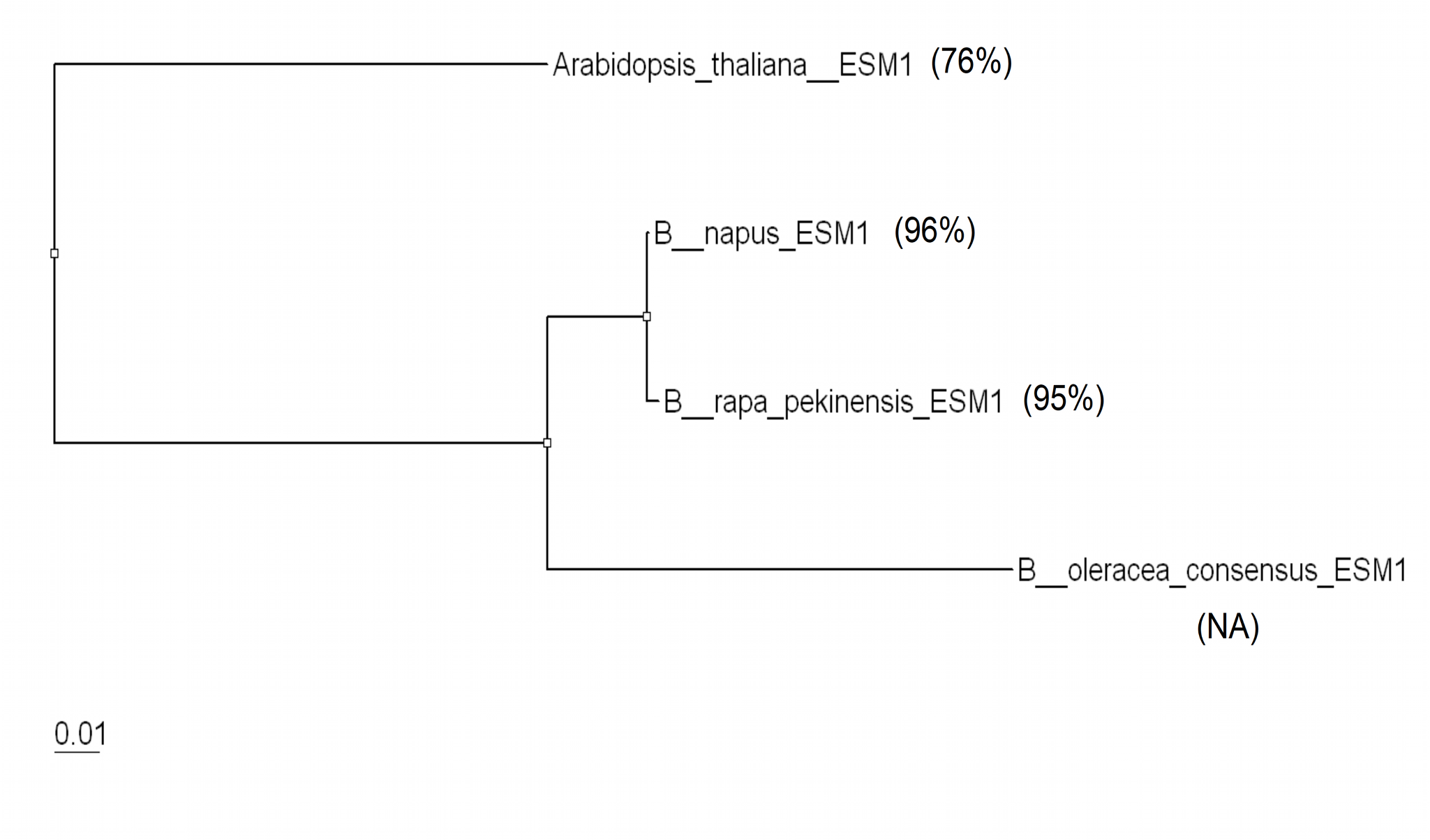

Supplement: Figure S1 — Phylogenetic tree of epithiospecifier modifier 1 (ESM1) co-factor associated with glucosinolate hydrolysis based on the amino acid sequences deduced from the isolated cDNA sequences. Brassica oleracea consensus (cabbage, broccoli, and cauliflower), Brassica rapa ssp. perkinesis (ACO57702.1), Brassica napus (ACO57703.1), and Arabidopsis thaliana ESM1 (ABB90255.1) used to construct phylogenetic tree. The values in parenthesis are amino acid sequence similarity with B. oleracea consensus by using NCBI BLAST search. The tree was constructed using Clustal W2 (http://www.ebi.ac.uk/Tools/clustalw2/). (TIF) [file pone.0077127.s001.tif]

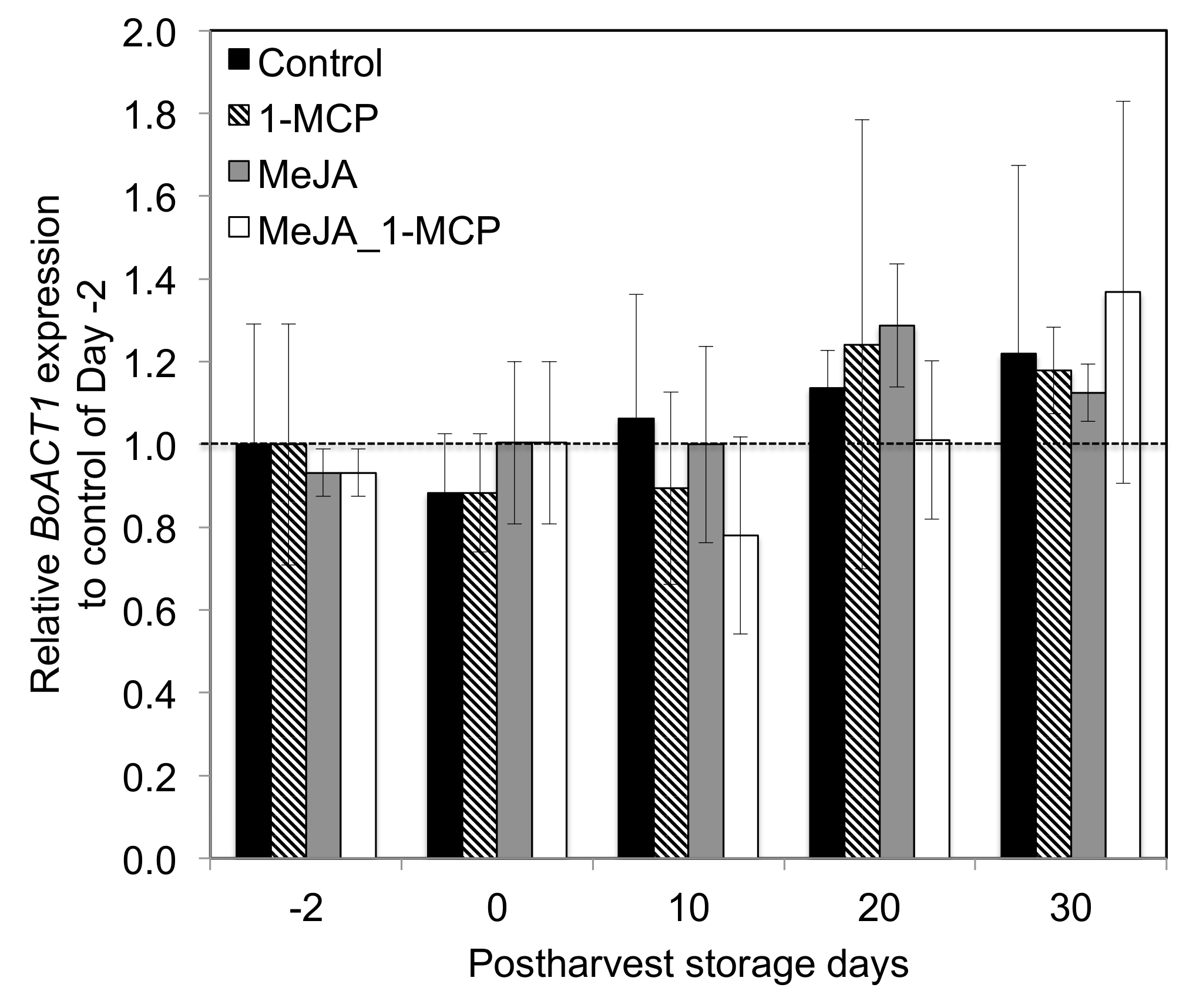

Supplement: Figure S2 — Transcript abundance of BoACT1 at two days before harvest, at harvest, and during post-harvest storage at 4 °C. (TIF) [file pone.0077127.s002.tif]

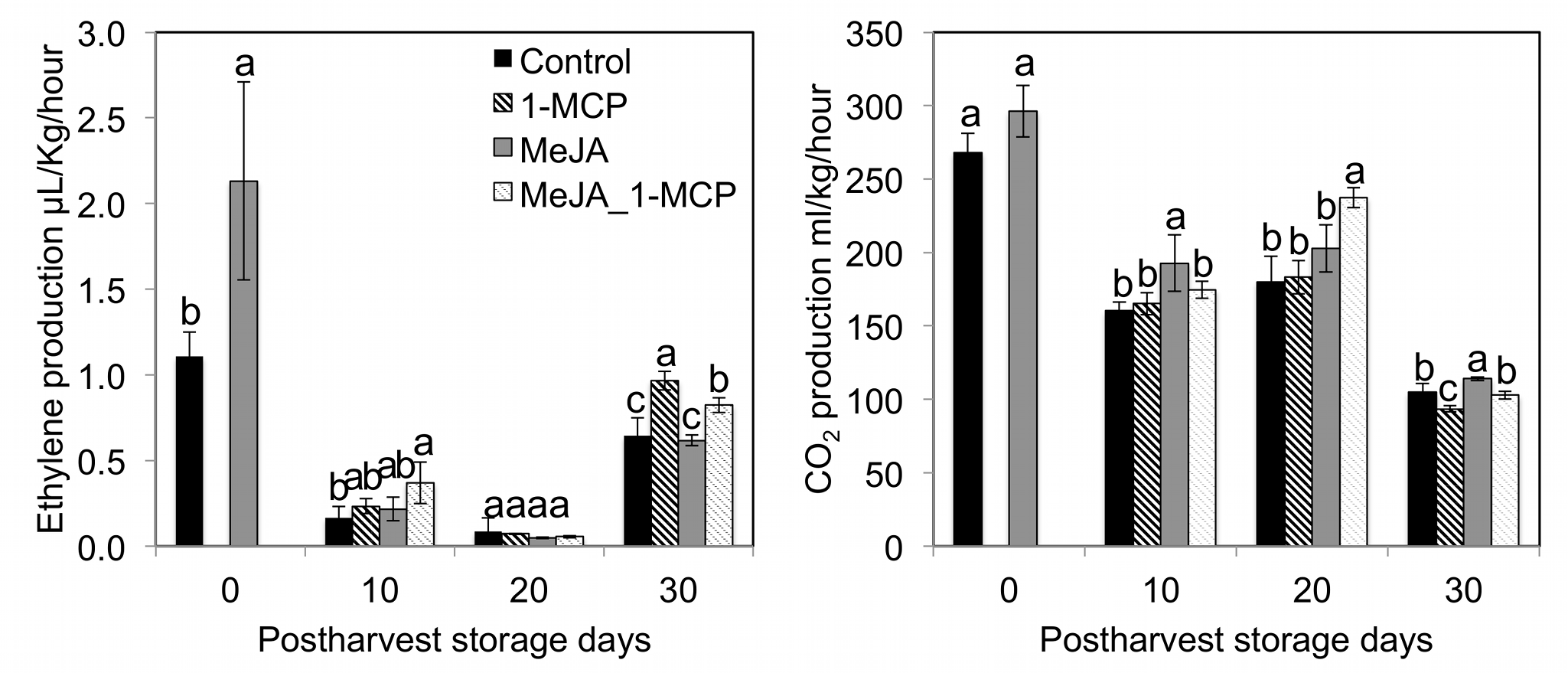

Supplement: Figure S3 — Effects of pre-harvest MeJA and post-harvest 1-MCP treatments on ethylene production and respiration rate of broccoli florets at harvest and at 10, 20, and 30 days of post-harvest storage at 4 °C. Different letters indicate significant differences among treatments based on Fisher’s LSD test at P ≤ 0.05. Mean ± SD (n=3). (TIF) [file pone.0077127.s003.tif]

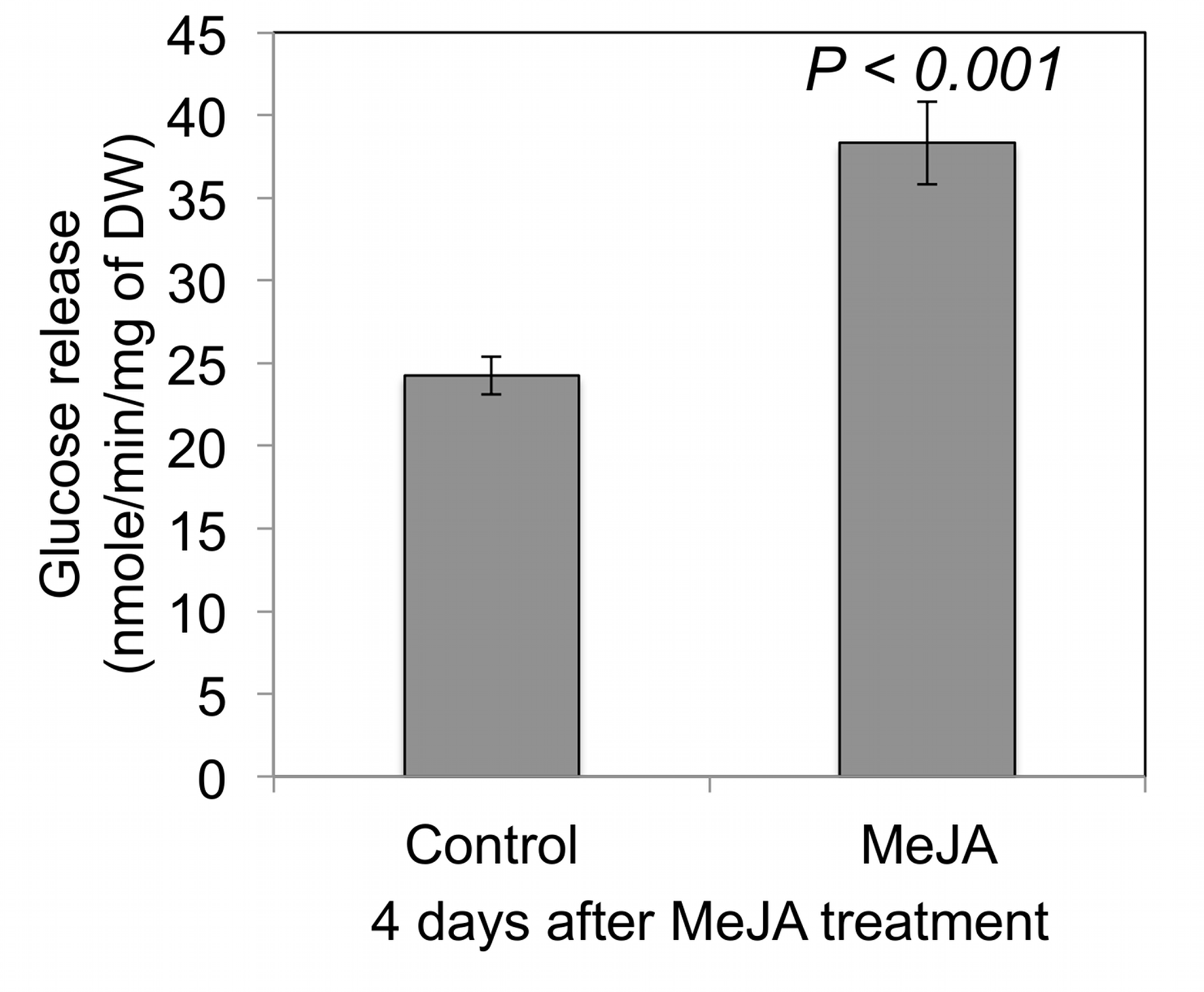

Supplement: Figure S4 — Effect of MeJA treatment on broccoli floret myrosinase activity at harvest. Student’s T-test was conducted to determine significance. Mean ± SD (n=3). (TIF) [file pone.0077127.s004.tif]

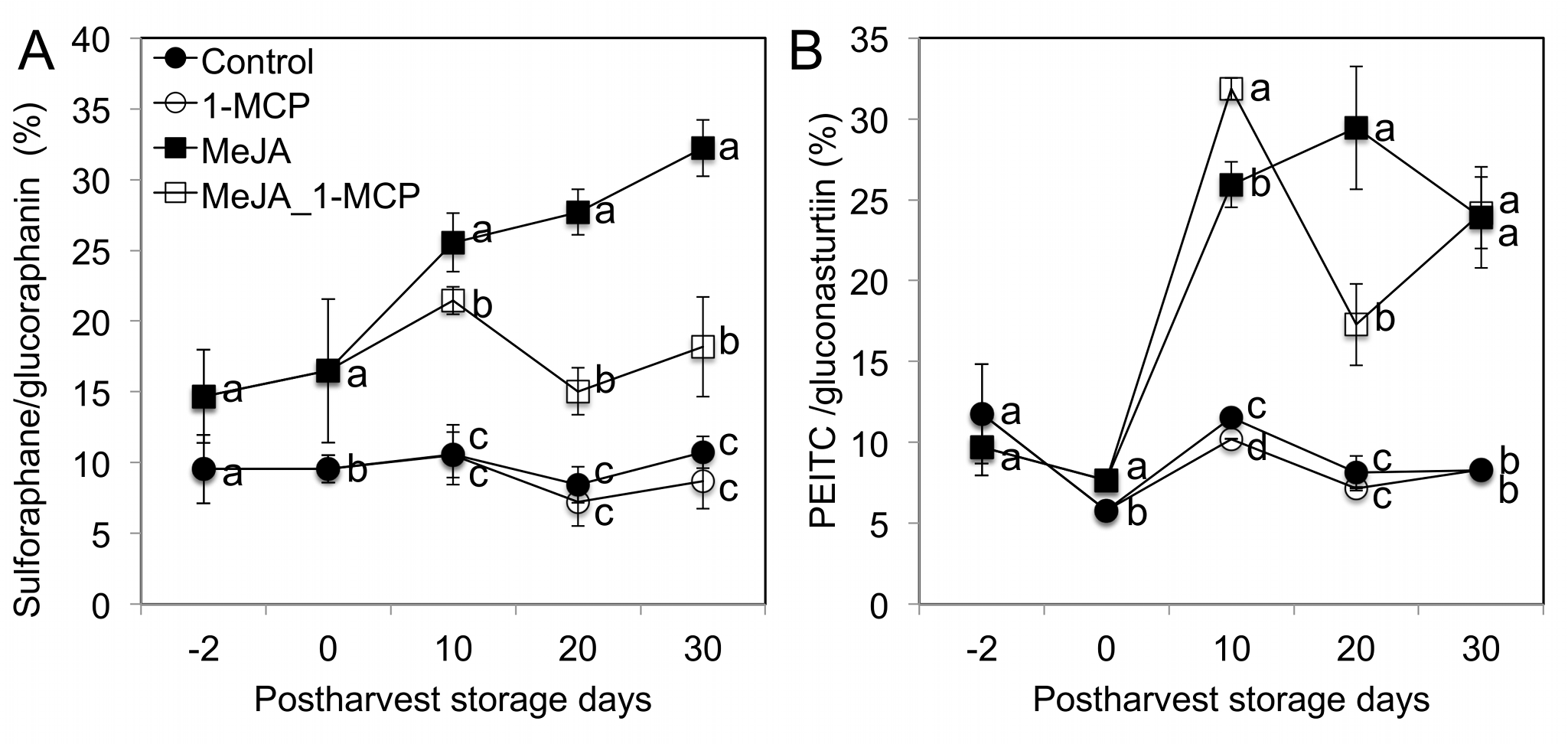

Supplement: Figure S5 — Sulforaphane and phenethyl isothiocyanate (PEITC) conversion from glucoraphanin and gluconasturtiin at two days before harvest, at harvest, and during post-harvest storage at 4 °C. Different letters indicate significant differences among treatments based on Fisher’s LSD test at P ≤ 0.05. Mean ± SD (n=3). (TIF) [file pone.0077127.s005.tif]
